# Acute‐Care Utilization and Cost Offsets Associated With Language‐Concordant, Pharmacist‐Integrated Care Management Among High‐Need, High‐Cost Adults

**DOI:** 10.1111/1475-6773.70127

**Published:** 2026-05-11

**Authors:** Sasha R. Sioni, Lesley Manson, Nicholas Arledge, Janice Yanez, Mohammad Al Mouslmani

**Affiliations:** ^1^ College of Health Solutions, Arizona State University Phoenix Arizona USA; ^2^ Miami Herbert Business School, University of Miami Coral Gables Florida USA; ^3^ Department of Medical Education Herbert Wertheim College of Medicine, Florida International University Miami Florida USA; ^4^ Merit Health River Region Vicksburg Mississippi USA; ^5^ Nicklaus Children's Hospital Miami Florida USA; ^6^ Nicole Wertheim College of Nursing & Health Sciences, Florida International University Miami Florida USA; ^7^ Department of Hospital Medicine Yale New Haven Hospital New Haven Connecticut USA

**Keywords:** emergency department visits, high‐cost adults, high‐need, hospital admissions, language‐concordant care management, pharmacist‐integrated care management, return on investment, safety‐net clinics

## Abstract

**Objective:**

To estimate utilization and economic effects of a language‐concordant, pharmacist‐integrated care‐management program and examine equity by language and insurance.

**Study Setting and Design:**

We conducted a retrospective cohort study emulating a target trial across four Phoenix safety‐net clinics (March 2022–September 2023) using inverse probability weighting and doubly robust Poisson difference‐in‐differences.

**Data Sources and Analytic Sample:**

We linked electronic health records, payer claims, health information exchange admission‐discharge‐transfer alerts, and pharmacist logs for 526 high‐need, high‐cost adults. Enrollees (*n* = 263) were matched 1:1 to otherwise eligible usual‐care comparators (*n* = 263) from the same clinics and calendar period.

**Principal Findings:**

Within 60 days, hospital admissions (average marginal effect [AME], −0.44; 95% confidence interval [CI], −0.60 to −0.28; incidence‐rate ratio [IRR], 0.50 [0.29–0.86]) and emergency department visits (AME, −0.16; 95% CI, −0.27 to −0.05; IRR, 0.47 [0.28–0.77]) were lower. Using standardized 2024 national unit costs and a $470 program cost, estimated net savings were $6421 per enrollee (return on investment [ROI], 13.66:1). Spanish‐preferring subgroup estimates were directionally similar, and formal interaction tests were not statistically significant.

**Conclusions:**

In safety‐net clinics, this language‐concordant, pharmacist‐integrated multicomponent program was associated with lower short‐term hospital use and substantial near‐term standardized cost offsets.

## Callout Box

- What is known on this topic.
  ○ High‐need, high‐cost adults account for a disproportionate share of U.S. spending, and language discordance is associated with higher readmission and emergency department revisit risk.
  ○ Pharmacist‐supported transitions of care can reduce readmissions.
  ○ Communication barriers after discharge can impede comprehension, medication management, and follow‐up.
- What this study adds.
  ○ Compared with similar patients receiving usual care, bilingual pharmacist‐integrated care management reduced 60‐day hospital admissions by 0.44 and emergency department visits by 0.16 per participant.
  ○ Using standardized 2024 national unit costs and a $470 program cost, estimated net savings were $6421 per participant (return on investment, 13.66:1).
  ○ Spanish‐preferring participants showed a similar pattern of benefit, but interaction tests did not show clear evidence of effect modification by language preference.

## 1 Introduction

High‐need, high‐cost adults—the top 5% of users—account for nearly half of U.S. health spending, straining safetynet systems as national spending reached $4.9 trillion in 2023 [1, 2]. These pressures intersect with communication barriers: 27.6 million U.S. residents (≥ 5 years) had limited English proficiency (LEP) in 2023, a status consistently linked to access and quality gaps [3]. LEP is prevalent in safety—net care and co‐occurs with social and coverage vulnerabilities, increasing risk for avoidable acutecare use [3].

Growing evidence links language barriers to higher short‐term acute‐care use. Pooled analyzes show language discordance is associated with higher adjusted 30‐day readmissions and emergency department (ED) revisits in adults and children [4]. In a large Upper Midwest multihospital cohort, adults with LEP had higher odds of irregular emergency department (ED) departures and more 72‐h and 7‐day returns, underscoring safety and cost risks [5]. Reviews find language‐concordant care or high‐quality interpreter services improve comprehension, satisfaction, and clinical outcomes, though effects vary by context and modality [6]. Despite progress, few evaluations of language‐concordant, pharmacist‐integrated care‐management models report economic endpoints (costs, budget impact, return on investment [ROI]); most emphasize readmissions, and cost analyzes—when present—are narrow [7, 8].

We address these gaps via a secondary analysis of a bilingual, pharmacist‐integrated multicomponent care‐management program across four safety‐net clinics. Guided by Andersen's Behavioral Model and Levesque's access framework, we hypothesized that enrollment in this multicomponent program—combining bilingual pharmacist counseling and medication reconciliation, structured social‐risk screening/navigation, and coordinated postdischarge follow‐up—would reduce near‐term hospital admissions and ED visits and be cost saving [9, 10]. Our objective was to quantify short‐horizon utilization and economic effects and examine equity‐relevant heterogeneity by language and insurance [7, 8, 11].

## 2 Methods

### 2.1 Design, Setting, and Oversight

We conducted a retrospective cohort emulating a target trial in four Phoenix, Arizona, safety‐net primary care clinics (March 1, 2022–September 30, 2023). The manuscript adheres to STROBE, RECORD, and CHEERS 2022 [12, 13, 14]. The institutional review board (IRB) approved the study ([IRB protocol number blinded for review]). Because analyzes used fully de‐identified data, the Board granted a waiver of informed consent; enrolled participants provided informed consent for patient‐reported outcomes (PROs).

### 2.2 Participants, Intervention, Comparators, and Matching

Adults (≥ 18 years) were eligible with ≥ 2 hospital admissions or ≥ 4 emergency department (ED) treat‐and‐release visits in the prior 12 months and deterministic linkage across data sources. Enrollees were approached within 48 h of index discharge. The multicomponent intervention combined bilingual pharmacist counseling and medication reconciliation with structured social‐risk screening/navigation and coordinated postdischarge follow‐up. Matched comparators were drawn from the broader eligible usual‐care pool in the same clinics and calendar period—not only patients who declined or did not provide consent—and contributed electronic health record (EHR), claims, and health information exchange (HIE) outcomes without structured care‐management exposure. EuroQol 5‐Dimension 5‐Level (EQ‐5D‐5L) utility and Net Promoter Score (NPS) were collected at baseline and 60 days in both arms.

Of 684 eligible patients, 512 were contacted and 279 consented. We drew a 1:1 matched comparator sample from the eligible usual‐care pool using nearest‐neighbor matching on age, sex, primary language, race/ethnicity, payer, Social Vulnerability Index [SVI], baseline 12‐month admission and ED counts, clinic, and index month; observations outside common support were trimmed before propensity‐based weighting (Table S3; Figure S2).

After excluding 16 consented participants lacking baseline patient‐reported outcomes, the analytic sample comprised 263 enrollees and 263 matched comparators (*N* = 526; 31,560 person‐days). All standardized mean differences were < 0.10 after IPTW (Table S3 and Figure S2), with a small residual EQ‐5D‐5L imbalance (SMD = 0.046) adjusted in all models. PROs were directly observed for 263 enrollees and 224 comparators; 39 comparator records were carried via multiple imputation (Table S7).

### 2.3 Data Sources, Linkage, and Encounter Reconciliation

We linked electronic health records (EHRs), payer claims, regional health information exchange (HIE) admission‐discharge‐transfer (ADT) alerts, and pharmacist care‐management logs using hashed member‐ID combinations; a 5% audit verified linkage accuracy. EHR addresses were geocoded to Census tracts to assign CDC Social Vulnerability Index (SVI) [15]. Symmetric ±24‐h cross‐feed deduplication collapsed duplicate inpatient or ED encounters (Claims > EHR > HIE precedence); claims backfill added 12 inpatient episodes in comparators and 0 in enrollees over 60 days. ADT alerts were an outcome source, not an intervention [16, 17]. Reconciliation details are in Table S1; deduplication‐related “ROI credits” were excluded.

### 2.4 Measures: Utilization Outcomes, PROs, and Effect Modifiers

Primary outcomes were all‐cause inpatient admissions and treat‐and‐release ED visits in 60‐day pre (−60, 0] and post (0, 60] windows. Table S2 provides code sets and Appendix S2 the codebook. Seven‐day endpoints appear in Appendix S1; 30‐ and 60‐day outcomes are in Table 1. PROs were EQ‐5D‐5L utility and NPS, collected at baseline and ≈60 days with identical procedures across arms. Observed PRO data were available for 263 enrollees and 224 comparators; 39 comparator records remained in the analytic denominator via multiple imputation, with missingness concentrated in comparators. EQ‐5D‐5L scoring followed EuroQol guidance; NPS was interpreted descriptively [16, 17]. Race, ethnicity, primary language, and insurance were prespecified confounders/modifiers, and SVI was prespecified for heterogeneity analyzes; race/ethnicity reporting followed JAMA guidance, with “Other/Multiracial” aggregation and small‐cell suppression [18, 19].

**TABLE 1:** Primary utilization and economic outcomes at 30‐ and 60‐days postindex.

Horizon	Hospital admissions, IRR (95% CI)	Admissions AME, per participant	ED visits, IRR (95% CI)	ED visits AME, per participant	Gross standardized cost offset per enrollee	Program cost per enrollee	Net standardized savings per enrollee	ROI
30 days	0.21 (0.13–0.34)	−0.368	0.52 (0.33–0.81)	−0.118	$5749.32	$470.00	$5279.32	11.23:1
60 days	0.50 (0.29–0.86)	−0.44	0.47 (0.28–0.77)	−0.16	$6891.14	$470.00	$6421.14	13.66:1

*Note:* IRRs < 1.00 indicate lower utilization among enrollees than among weighted comparators. AMEs were estimated from doubly robust Poisson difference‐in‐differences models. Gross standardized cost offsets apply 2024 national unit costs to model‐estimated reductions in hospital admissions and treat‐and‐release ED visits; net standardized cost offsets subtract the $470 program cost per enrollee. Economic values represent standardized near‐term cost offsets, not realized payer‐specific expenditures.

Abbreviations: AME, average marginal effect; CI, confidence interval; ED, emergency department; IRR, incidence‐rate ratio; ROI, return on investment; USD, U.S. dollars.

### 2.5 Causal and Statistical Analysis

We emulated a target trial [20], anchoring time zero at discharge: enrollees at the outreach‐triggering discharge; comparators at the nearest qualifying discharge in the same clinic/week (±3 d; ties earlier). Outcomes accrued during days 1–60 after index discharge; follow‐up was censored at death or out‐migration, with no subsequent enrollment. Gradient‐boosted propensity scores yielded stabilized inverse probability of treatment weighting (IPTW) with 1st/99th‐percentile trimming and standardized mean differences < 0.10 (Table S3; Figure S2). We estimated the 60‐day treatment effect on the treated via doubly robust Poisson difference‐in‐differences (DiD) with clinic fixed effects and Huber–White standard errors, reporting incidence‐rate ratios (IRRs) and average marginal effects (AMEs) with 95% confidence intervals (CIs). Rare missing covariates were handled with chained‐equations multiple imputation (*m* = 20) and pooled via Rubin's rules.

**FIGURE 1:**
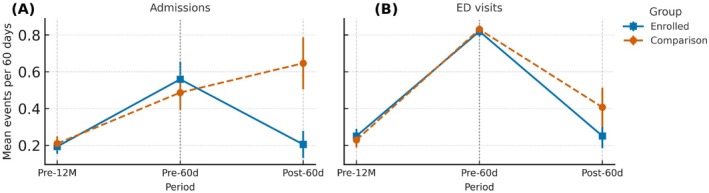
Mean acute‐care events per 60 days across pre‐ and postindex windows. (A) Shows all‐cause inpatient admissions and (B) shows treat‐and‐release ED visits for enrollees and comparison participants across three summary windows: The 12 months before index discharge (displayed on the same per‐60‐day scale as the other windows), the 60 days before index discharge, and the 60 days after index discharge. Distinct line/marker styles denote the two groups; lines connect group‐specific mean event counts for readability only. This figure is descriptive; formal preperiod lead estimates and joint Wald tests for the parallel‐trends assumption are reported in Table S11, and the supplemental event‐study‐style panels for hospital admissions and ED visits are presented in Figure S3. ED, emergency department.

Figure 1 summarizes mean acute‐care events across pre‐ and postindex windows. Pretrend diagnostics, multiple‐comparison corrections (Benjamini–Hochberg for interaction tests, Holm for prespecified subgroups by language, insurance, and social vulnerability index) [21, 22, 23]. *E*‐values for the primary incidence‐rate ratios and an a priori negative‐control outcome (7‐day outpatient preventive visits) [24, 25], and entropy‐balancing robustness checks are provided in the supplement [12, 13, 14, 21, 22, 26, 27, 28, 29]. Enrollment‐model performance was moderate (AUC, 0.70–0.74; Brier, 0.182–0.188; Tables S3, S4, S11, S12).

### 2.6 Economic Methods

We conducted a 60‐day standardized cost‐offset analysis using 2024 national unit costs for inpatient stays (HCUP Fast Stats and National Inpatient Sample) [30, 31], and treat‐and‐release ED visits [32], repriced to 2024 U.S. dollars via the Consumer Price Index Medical Care series [33, 34]. We report weighted mean standardized 60‐day cost differences, net monetary benefit, and return on investment (ROI) = (standardized costs avoided − program cost)/program cost; the program cost was $470 per enrollee. Thirty‐day estimates used the same framework. Unit costs were varied by ±20% while holding the program cost constant. Claims‐paid or severity‐adjusted sensitivity analyzes were not feasible because patient‐level adjudicated paid amounts and a harmonized severity proxy were unavailable in the linked data. Uncertainty used nonparametric bootstrap (2000 replications) and probabilistic sensitivity analysis (Appendix S1) [35].

## 3 Results

Consented participants were slightly younger, more often Spanish‐preferring, and from more vulnerable neighborhoods than the broader eligible pool; residual selection bias is mitigated but not eliminated (Figure S1; Table S8).

### 3.1 Data‐Quality Reconciliation

Symmetric ±24 h cross‐feed deduplication with claims backfill added +12 comparison‐arm admissions (none in enrollees) over 60 days (Table S1).

### 3.2 Primary Utilization Outcomes

Table 1 and Figure 1 summarize 30‐ and 60‐day outcomes for admissions and ED visits. Under stabilized IPTW, doubly robust Poisson difference‐in‐differences models with clinic fixed effects and a log(60‐day) exposure offset showed lower 60‐day hospital admissions (IRR = 0.50, 95% CI [0.29, 0.86]; AME = −0.44 admissions per participant) and ED visits (IRR = 0.47, 95% CI [0.28, 0.77]; AME = −0.16 visits per participant) than weighted comparators. Thirty‐day estimates were similar (Table 1). Formal pretrend diagnostics supported parallel trends (Wald tests: admissions *χ* ^2^(2) = 0.58, *p* = 0.75; ED visits *χ* ^2^(2) = 1.21, *p* = 0.55; Figure 1, Table S11). The admissions *E*‐value was 3.44 for the point estimate and 1.60 for the upper CI limit (0.86).

### 3.3 Subgroup and Interaction Analyzes

**FIGURE 2:**
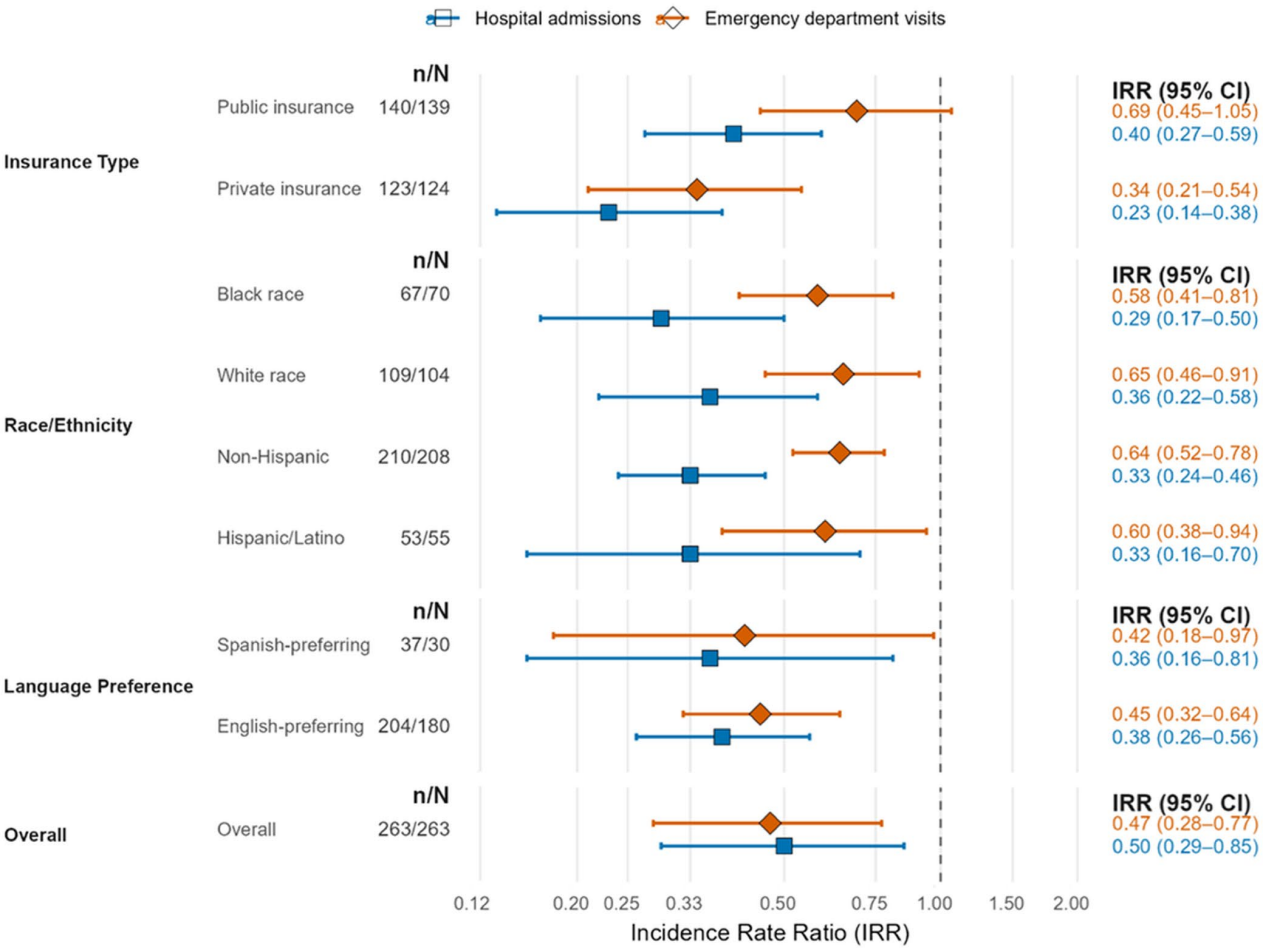
Equity‐stratified incidence‐rate ratios for 60‐day acute‐care utilization (hospital admissions and emergency‐department visits). Forest plot of adjusted incidence‐rate ratios (IRRs) by subgroup (insurance type, race/ethnicity, language preference, and overall) for all‐cause hospital admissions and treat‐and‐release emergency‐department visits at 60 days. Squares represent hospital‐admission IRRs, and diamonds represent emergency‐department‐visit IRRs; horizontal bars show 95% confidence intervals; the vertical reference line marks IRR = 1.00 on the log‐scale *x*‐axis. The right‐hand columns report subgroup‐specific IRRs with 95% confidence intervals; *n*/*N* indicates enrolled/comparison counts. Estimates were obtained from stabilized inverse probability of treatment weighting–weighted, doubly robust Poisson difference‐in‐differences models with clinic fixed effects and Huber‐White standard errors (SEs). CI, confidence interval; DiD, difference‐in‐differences; ED, emergency department; IRR, incidence‐rate ratio; IPTW, inverse probability of treatment weighting; MI, multiple imputation; *n*/*N*, enrollee/comparator counts; SE, standard error.

Among Spanish‐preferring participants, subgroup IRRs suggested similar benefit (admissions, 0.36 [95% CI, 0.16–0.81]; ED, 0.42 [0.18–0.97]), but interaction tests showed no clear effect modification by language preference. Across race/ethnicity, sex, and prior ED‐use strata, IRRs generally mirrored overall effects; estimates for Other/multiracial and zero baseline ED use marginally crossed 1.00. Intersectional analyzes (Hispanic/Latino × Spanish‐preferring × Medicaid) suggested borderline synergy (synergy index = 1.42; 95% CI, 1.01–1.93) [36, 37]. See Table S6 and Figure 2 for subgroup effects, Table S9 for equity‐weighted results, and Table S10 for detectable IRRs (80% power).

Patient‐reported outcomes. PROs were secondary endpoints. In the primary analysis with multiple imputation across the matched cohort (*N* = 526), EQ‐5D‐5L improved more for enrollees over 60 days (ΔDiD = +0.08; 95% CI, 0.06–0.10; *p* < 0.001). All 39 missing PRO records occurred among comparators; observed‐case and multiply imputed estimates were concordant (Appendix S1, Tables A1–A2), though secondary PRO findings remain more vulnerable to differential nonresponse than primary utilization outcomes. Language‐by‐time interactions were not statistically significant, with similar descriptive trends among English‐ and Spanish‐preferring enrollees (Figure S5).

### 3.4 Robustness and Diagnostics

Sensitivity analyzes—entropy balancing, augmented inverse‐probability‐weighted difference‐in‐differences, 5th/95th‐percentile trimming, multiple‐imputation diagnostics, a negative‐control outcome, and Rosenbaum bounds—produced convergent estimates (e.g., entropy‐balancing admissions IRR = 0.41; 95% CI, 0.26–0.66). Goodman–Bacon decomposition yielded a single strictly positive weight (*ω* = 1.00), and the deviance/df ratio of approximately 1.1 supported the Poisson specification with robust variance (Figure S3; Table S12).

### 3.5 Economic Outcomes

Using 2024 repriced standardized national unit costs and a $470 program cost, weighted mean standardized 60‐day costs were $3598 for enrollees versus $9560 for comparators (Δ = −$5962; 95% CI, −$8455 to −$3621) [30, 31, 32, 33, 34]. Gross standardized cost offsets were $6891 at 60 days and $5749 at 30 days; net standardized cost offsets were $6421 and $5279 per enrollee, with ROI of 13.66:1 and 11.23:1 (Table 1). The incremental cost‐effectiveness ratio was dominant at both horizons, and probabilistic sensitivity analysis indicated ≥ 98% probability of cost‐effectiveness at ≤ $20,000 per admission avoided. These estimates reflect standardized near‐term hospital cost offsets, not realized payer‐specific expenditures (Figure S4, Table S5, Appendix S1).

## 4 Discussion

In this target‐trial emulation across four safety‐net clinics, enrollment in a language‐concordant, pharmacist‐integrated care‐management program was associated with lower 60‐day hospital use than weighted usual care, corresponding to 0.44 fewer admissions and 0.16 fewer emergency department visits per participant (IRRs, 0.50 and 0.47). Conservative 2024 USD standardized costing showed $6421 net savings per enrollee (ROI = 13.66:1) over 60 days. EQ‐5D‐5L also improved over 60 days. Effects were directionally similar among Spanish preferring participants; patient‐reported outcomes improved without differential language gains. Diagnostics were consistent with key model assumptions, but residual confounding and outcome misclassification cannot be excluded.

### 4.1 Relation to Prior Literature

Our findings align with prior work linking language barriers to higher readmissions and ED revisits and showing that language‐concordant care or high‐quality interpreter services can improve communication and some clinical outcomes [4, 5, 6]. Because this bundled intervention was not designed to compare language‐support models directly, subgroup findings are hypothesis‐generating and do not establish superiority of bilingual delivery over interpreter‐supported approaches. We quantify near‐term economic value through an equity lens, addressing gaps in evaluations of multicomponent care‐management models in safety‐net settings [7, 8]. The 60‐day admission reduction (AME = −0.44) exceeds many effects reported for general transition programs [38].

### 4.2 Programmatic and Policy Implications

For safety‐net systems, the implication is to embed language‐concordant staffing or interpreter capacity within reliable transitions workflows, not simply add bilingual staff. In this study, that component operated within a bundle of pharmacist counseling, medication reconciliation, structured social‐risk screening/navigation, and coordinated postdischarge follow‐up. Equity‐weighted net monetary benefit was positive across racial and ethnic groups, suggesting distributive value alongside savings. These findings support investment in coordinated transitions infrastructure while leaving the most efficient component mix unsettled [4, 5, 6, 7, 8, 25, 29, 39].

### 4.3 Strengths and Innovations

Strengths include integrating linked electronic health record, claims, health‐information‐exchange, and pharmacist data with doubly robust estimation, absolute‐effect reporting, and standardized short‐horizon economic translation. Decile‐based calibration of the enrollment model (Table S13) enhances transparency about selection and generalizability. Our focus on concordance is supported by evidence that language‐concordant discharge education and follow‐up improve comprehension and reduce readmissions [36, 40, 41, 42, 43]. Pharmacist‐supported transitions—counseling and medication reconciliation—also reduce 30‐day readmissions [38]. Linking EHR, claims, and real‐time ADT alerts improves encounter capture and supports equity‐stratified reporting by language, race/ethnicity, and coverage [16, 44, 45]. This pharmacist component also aligns with evidence favoring multicomponent discharge interventions over single‐contact approaches [38].

### 4.4 Limitations

Several limitations temper interpretation. First, despite target‐trial emulation, weighting, clinic adjustment, preperiod diagnostics, and convergent sensitivity analyzes, residual confounding and outcome misclassification cannot be excluded. Second, the bundled design does not isolate the independent contribution of language‐concordant delivery relative to professional interpreter‐supported care or other program components. Third, although the PRO collection protocol was the same in both arms, all missing PRO records occurred among comparators, and exploratory diagnostics linked missingness to post‐index ED use; accordingly, PRO results are more vulnerable to differential nonresponse than the primary utilization outcomes, despite concordant multiply imputed and observed‐case estimates. Fourth, the economic analysis estimates standardized national short‐term cost offsets rather than realized payer‐specific expenditures. Fifth, subgroup precision was limited, so the Spanish‐preferring estimates support a similar pattern of benefit rather than a definitively larger language‐specific effect. Finally, generalizability beyond four safety‐net clinics, other language groups, and differently financed delivery systems remains uncertain.

### 4.5 Conclusions and Next Steps

In safety‐net clinics, a language‐concordant, pharmacist‐integrated multicomponent care‐management program was associated with lower 60‐day hospital use and near‐term standardized cost offsets ($6421 net per enrollee; ROI, 13.66:1). These findings suggest that multicomponent transitions support can reduce short‐term acute‐care use while strengthening financial sustainability in safety‐net settings, but residual selection bias and possible unmeasured confounding limit causal inference [4, 5, 46]. Next steps are to test durability and payer‐specific budget impact in pragmatic multisite studies with prespecified heterogeneity analyzes, compare bilingual staffing with interpreter‐supported models, extend the model to additional languages and payers, and embed implementation and patient‐reported metrics to track fidelity, experience, and unintended effects.

## Funding

The authors have nothing to report.

## Ethics Statement

The institutional IRB approved the study (STUDY00021390). Because data were fully de‐identified, the board granted a waiver of informed consent; enrolled participants provided consent for PROs.

## Conflicts of Interest

The authors declare no conflicts of interest.

## Supporting information


**Appendix S1:** hesr70127‐sup‐0001‐AppendixS1.docx.

**Appendix S2:** hesr70127‐sup‐0002‐AppendixS2.docx. **Table S2**. Variable definitions, source fields, coding logic, and validation sources.

**Appendix S3:** STROBE statement—checklist of items that should be included in reports of observational studies.

**Appendix S4:** CHEERS 2022 checklist.

**Appendix S5:** The RECORD statement—checklist of items, extended from the STROBE statement, that should be reported in observational studies using routinely collected health data.

**Figure S1:** Assessment of cohort flow from screening to analysis, four Phoenix safety‐net clinics (March 1, 2022–September 30, 2023). Flow of participants from initial screening through inclusion in analyzes. Of 1452 records screened, 684 met high‐need, high‐cost (HNHC) criteria; 512 were contacted and invited; 106 declined and 127 provided no consent; 279 consented. Sixteen had incomplete baseline patient‐reported outcomes (PROs), yielding 263 enrolled participants. A matched comparison group (*n* = 263) was selected, and both groups were analyzed with no loss to follow‐up (total *N* = 526). Side boxes enumerate exclusion reasons at each stage (ineligible: < 2 acute encounters; unreachable: ≥ 3 attempts; declined/no consent; incomplete baseline PROs). HNHC was defined a priori as ≥ 2 inpatient admissions or ≥ 4 treat‐and‐release emergency department (ED) visits in the prior 12 months. Matched comparison participants were drawn from the broader eligible usual‐care pool in the same clinics and calendar period, not only from contacted patients who declined or did not provide consent; they were identified from the same source systems, selected 1:1 before IPTW, and screened to confirm no structured care‐management or pharmacist‐counseling exposure. Abbreviations: ADT, admission–discharge–transfer; ED, emergency department; HNHC, high‐need, high‐cost; IPTW, inverse probability of treatment weighting; *n*, number; PROs, patient‐reported outcomes.
**Figure S2:** Standardized mean differences before and after weighting for baseline covariates (target |SMD| < 0.10). Points show preweighting standardized mean differences (SMDs) and diamonds show postweighting SMDs after stabilized inverse probability of treatment weighting (IPTW) with 1st/99th‐percentile trimming and clinic fixed effects as specified in Methods. The solid vertical line marks 0; dashed lines mark the a priori balance threshold |SMD| < 0.10. Covariates include demographics (age, sex), race/ethnicity (White, Black, Hispanic/Latino, Other/multiracial), primary language (English, Spanish, Arabic, Vietnamese, Other), insurance (Medicare/Medicaid), education, income bands, prior utilization (12‐month and 60‐day hospital admissions and ED visits), neighborhood Social Vulnerability Index (SVI), and baseline patient‐reported measures (EQ‐5D‐5L utility, Net Promoter Score [NPS]). For continuous variables, SMDs were calculated as the absolute mean difference divided by the pooled standard deviation; for binary indicators, SMDs were calculated using the standard binary form; multicategory variables were represented by level‐specific indicators.
**Figure S3:** Event‐study‐style summary panels for hospital admissions and emergency‐department visits. These supplemental panels summarize manuscript‐reported preperiod lead estimates and postindex incidence‐rate ratios for hospital admissions and emergency‐department visits, comparing enrollees with weighted usual‐care comparators. Figure 1 provides a descriptive summary of mean acute‐care use across pre‐ and postindex windows, and Table S11 reports the formal preperiod lead coefficients and joint Wald tests for both outcomes. Points indicate incidence‐rate ratios, error bars indicate 95% confidence intervals, and the dashed reference line marks the null (IRR = 1.00). Abbreviations: CI, confidence interval; ED, emergency department; IRR, incidence‐rate ratio.
**Figure S4:** Step‐wise 60‐day standardized net savings per enrollee (2024 USD) and ROI. Bars show cumulative 60‐day standardized net savings per enrollee at each reconciliation step: (A) baseline effects using legacy 2019 unit costs; (B) symmetric duplicate‐episode reconciliation with claims backfill applied to both study arms; (C) update of the inpatient unit cost to the HCUP 2021 national mean; (D) update of the treat‐and‐release emergency‐department unit cost to the HCUP 2021 national mean; and (E) repricing of 2021 unit costs to 2024 U.S. dollars using the annual‐average Consumer Price Index for All Urban Consumers: Medical Care series (CPIMEDNS). The final bar (E) corresponds to the analytic estimate reported in the manuscript (net savings = $6421.14; ROI = 13.66:1). ROI was calculated as (standardized costs avoided—program cost)/program cost; program cost was $470 per enrollee. Deduplication and claims backfill are shown only to reconcile source feeds and were not counted as independent value in the ROI calculation. Incremental step contributions and unit‐cost inputs are reported in Table S5.
**Figure S5:** Longitudinal patient‐reported‐outcome trends by language preference. One panel displays mean EQ‐5D‐5L utility scores, and the other displays mean Net Promoter Scores, at baseline and approximately 60 days after discharge for Spanish‐preferring (red) and English‐preferring (orange) enrollees. Error bars indicate 95% confidence intervals. Trends are shown for enrollees only. Abbreviations: CI, confidence interval; EQ‐5D‐5L, EuroQol 5 Dimension 5 Level; NPS, Net Promoter Score; PROs, patient‐reported outcomes.

**Table S1:** Duplicate encounter audit and reconciliation (symmetric cross‐feed deduplication + claims backfill) and impact on 60‐day admissions (*N* = 526).
**Table S2:** Variable definitions, code systems, and algorithms.
**Table S3:** Residual covariate imbalance after IPTW (postweight SMDs; *N* = 526).
**Table S4:** Predictive enrollment model (baseline‐only)
**Purpose**. Sensitivity analysis to characterize selection into enrollment; does not replace the primary IPTW/DiD causal analysis. Models used only baseline variables (age, sex, Spanish language indicator, public insurance, 12‐month admissions and ED visits, SVI). Reporting follows TRIPOD guidance emphasizing discrimination and calibration (AUC; calibration slope/intercept; decile‐based calibration).
**Table S5:** Step‐wise 60‐day savings and ROI (from dedup/backfill → unit‐cost updates → 2024 repricing).
**Table S6:** Incidence rate ratios for 60‐day outcomes across equity‐related subgroups.
**Table S7:** Baseline characteristics of enrolled care‐management patients and matched comparators before inverse‐probability weighting (*N* = 526).
**Table S8:** Baseline representativeness of enrollees relative to the broader eligible usual‐care pool.
**Table S9:** Equity‐weighted cost‐effectiveness results (60‐day horizon).
**Table S10:** Detectable incidence‐rate ratios for equity‐related interaction contrasts (60‐day hospital admissions*).
**Table S11:** Formal preperiod lead estimates and joint Wald tests for the parallel‐trends assumption.
**Table S12:** Goodman–Bacon decomposition of the DiD effect (two‐period design).
**Table S13:** Baseline characteristics (Post‐IPTW, ATT; Weighted Means and SDs).
**Table S14:** Decile‐based calibration (GBM model).

## Data Availability

The data that support the findings of this study are openly available in Open Science Framework (OSF) at https://osf.io/fvrph/, reference number DOI: 10.17605/OSF.IO/FVRPH.
