# Supplementary material for: Acute‐Care Utilization and Cost Offsets Associated With Language‐Concordant, Pharmacist‐Integrated Care Management Among High‐Need, High‐Cost Adults
Source: Health Serv Res. 2026 May 11;61:e70127. doi: 10.1111/1475-6773.70127 (PMC13160595; doi:10.1111/1475-6773.70127)
Supplement: Supplementary file 1 — Appendix S1: hesr70127‐sup‐0001‐AppendixS1.docx. [file HESR-61-0-s006.docx]

**Appendix S1**

**Return-on-Investment (ROI) Reconciliation and Supplemental Methods**

**Scope note.** This appendix covers ROI reconciliation, short-horizon economic translation, patient-reported outcome (PRO) denominator crosswalks, and exploratory comparator-only missingness diagnostic; causal-identification diagnostics are reported in Figure S3 and Tables S11–S12.

**Data governance, provenance, and linkage**

Data came from 4 sources: clinic electronic health records (EHRs), administrative claims, regional health-information exchange (HIE) admission-discharge-transfer (ADT) alerts, and pharmacist care-management/reconciliation logs. Deterministic linkage used person-level identifiers, including date of birth and payer/member identifiers, together with discharge timing when available. Linkage success rates were 100% for EHR-to-claims matches, 96.6% for ADT alerts, and 95.4% for pharmacist logs, yielding a composite match rate of 95.4% for the analytic cohort (N = 526). A 5% blinded clerical audit (n = 26) detected zero false-positive links and 2 missed matches, corresponding to a positive predictive value of 100% and an estimated false-negative rate <1%.

**Duplicate-encounter audit and claims backfill (symmetric; applied to both arms)**

To ensure one clinical episode per outcome, we applied a symmetric cross-feed ±24-hour deduplication rule: encounters appearing in multiple feeds were collapsed to 1 episode, with precedence claims→EHR→HIE for dates/codes. Claims backfill resolved feed lag by adding encounters that arrived after the initial extracts. Over the 60-day window, the net count change occurred for inpatient admissions only: +12 counted inpatient episodes in the comparison arm and 0 in the enrollee arm. The unadjusted absolute difference changed from −0.1179 to −0.1635 admissions per participant, and the unadjusted IRR changed from 0.6310 (53/84) to 0.5521 (53/96) (Table S1). In the model-adjusted analysis used for ROI, the average marginal effect (AME) was −0.44 admissions and −0.16 ED visits per participant; step-wise before/after counts and models are reported in Table S1.

**Unit-cost repricing (base year 2021; 2024 USD via CPIMEDNS)**

Inpatient admission: US $14,318 (Healthcare Cost and Utilization Project national mean cost per stay, base year 2021).

Treat-and-release emergency department (ED) visit: US $750 (HCUP Statistical Brief No. 311; base year 2021).

Inflation adjustment to 2024: medical-care component of the Consumer Price Index for All Urban Consumers (CPIMEDNS, not seasonally adjusted), using the annual-average 2024/2021 ratio (approximately 1.073).

Program delivery cost: US $470 per enrollee.

These inputs were used to translate utilization effects into standardized near-term cost offsets. For transparency, we report both 2021-USD values (base costs) and the repriced 2024-USD values.

**ROI decomposition**

After deduplication/backfill and 2021 unit-cost updates, gross 60-day savings were US $6,419.92 per participant; subtracting the US $470 program cost yielded net savings of US $5,949.92 and an ROI of 12.6594:1 (rounded 12.66:1). When repriced to 2024 USD via the CPIMEDNS annual-average 2024/2021 ratio, gross 60-day savings were US $6,891.14, net savings were US $6,421.14, and ROI was 13.66:1. The reconciliation steps (baseline → symmetric deduplication + claims backfill → updated 2021 inpatient and ED unit costs → repricing to 2024 USD) are tabulated in Table S5 and visualized in Figure S4; the main economic estimates exclude any “credit” for data cleaning and rely only on the cleaned dataset.

**Cost-effectiveness uncertainty summary**

The weighted mean standardized 60-day cost difference and the AME-based savings estimate address related but distinct estimands: the former summarizes total observed standardized costs after weighting, whereas the latter prices the model-estimated reductions in admissions and ED visits used for ROI. Accordingly, the 2 quantities are not expected to be identical. The incremental cost-effectiveness ratio was dominant at both 30 and 60 days, and probabilistic sensitivity analysis indicated at least 98% probability of cost-effectiveness at willingness-to-pay thresholds up to US $20,000 per admission avoided. These values reflect standardized near-term cost offsets rather than realized payer-specific expenditures.

**Seven- and 30-day economic results**

Using the same unit-cost framework and analysis specifications as the 60-day endpoints—national mean 2021 costs for inpatient admissions (US $14,318) and treat-and-release ED visits (US $750), repriced to 2024 USD via the CPIMEDNS annual-average 2024/2021 ratio, and a program cost of US $470 per enrollee—the shorter-horizon estimates were as follows.

**7 days:** admissions IRR = 0.15 (95% CI, 0.08–0.29), Δ admissions = −0.237 per participant; ED visits IRR = 0.47 (95% CI, 0.22–1.01), Δ ED = −0.038 per participant. Gross savings were US $3,674.95; after the program cost, net savings were US $3,204.95 with an ROI of 6.82:1 (2024 USD).

**30 days:** admissions IRR = 0.21 (95% CI, 0.13–0.34), Δ admissions = −0.368 per participant; ED visits IRR = 0.52 (95% CI, 0.33–0.81), Δ ED = −0.118 per participant. Gross savings were US $5,749.32; after the program cost, net savings were US $5,279.32 with an ROI of 11.23:1 (2024 USD).

These estimates use the same 2021 unit-cost sources and 2024-dollar repricing used for the 60-day analysis; the step-wise reconciliation parallels the 60-day workflow summarized in Table S5.

**Abbreviations:** ADT, admission-discharge-transfer; AME, average marginal effect; CI, confidence interval; CPIMEDNS, Consumer Price Index for All Urban Consumers: Medical Care; ED, emergency department; EHR, electronic health record; HCUP, Healthcare Cost and Utilization Project; HIE, health-information exchange; IRR, incidence-rate ratio; ROI, return on investment; USD, U.S. dollars.

**Patient-reported outcome denominator crosswalk and missing-data sensitivity**

Table A1 separates the matched cohort from the directly observed PRO subset and shows that observed-case weighted and multiple-imputation estimates were materially concordant for EQ-5D-5L and Net Promoter Score.

**Appendix S1, Table A1. PRO denominator crosswalk and sensitivity to missing-data handling**

| **Analysis population** | **Enrolled, n** | **Comparison, n** | **Total, n** | **EQ-5D-5L ΔDiD (95% CI)** | **Net Promoter Score ΔDiD (95% CI)** |
| --- | --- | --- | --- | --- | --- |
| Directly observed PRO subset | 263 | 224 | 487 | +0.08 (0.05–0.10) | +9.82 (8.47–11.18) |
| Multiply imputed analytic PRO cohort | 263 | 263 | 526 | +0.08 (0.06–0.10) | +9.90 (8.87–10.94) |

**Note.** The matched cohort was N = 526 (263 enrollees; 263 comparators). In the shared extract, directly observed PRO data were available for all 263 enrollees and 224 comparators; the remaining 39 comparator records were retained in the analytic PRO denominator and handled with multiple imputation. Observed-case estimates are IPTW-weighted sensitivity analyses; multiply imputed estimates are the primary analytic PRO results.

**Abbreviations:** DiD, difference-in-differences; EQ-5D-5L, EuroQol 5-Dimension 5-Level; IPTW, inverse probability of treatment weighting; PRO, patient-reported outcome.

**Deterministic low/high scenario analysis**

As a deterministic low/high scenario analysis, we varied both 2024 unit costs by ±20% while holding the US $470 program cost constant. Under the low-cost scenario, gross 60-day savings were US $5,512.91, net savings were US $5,042.91, and ROI was 10.73:1. Under the high-cost scenario, gross 60-day savings were US $8,269.37, net savings were US $7,799.37, and ROI was 16.59:1. Net savings remained positive across both scenarios.

**Exploratory comparator-only missingness diagnostic**

Because all missing PRO records occurred among comparators, exploratory missingness diagnostics were fit among comparison participants only. Table A2 reports the comparator-only logistic model for any PRO missingness and is provided as a transparency diagnostic; it does not replace the locked analytic PRO denominator or the primary multiply imputed estimates in Table A1.

**Appendix S1, Table A2. Exploratory comparator-only model for any PRO missingness**

| **Predictor** | **OR** | **95% CI** | **p value** |
| --- | --- | --- | --- |
| Intercept | 0.06 | 0.01–0.40 | .004 |
| Age (per year) | 1.03 | 1.01–1.05 | .003 |
| Female sex | 0.88 | 0.40–1.94 | .753 |
| Spanish preference | 1.12 | 0.37–3.41 | .838 |
| Medicare/Medicaid insurance | 1.04 | 0.50–2.17 | .922 |
| Social Vulnerability Index | 0.52 | 0.07–4.09 | .531 |
| Admissions in prior 12 months | 0.71 | 0.51–0.98 | .039 |
| ED visits in prior 12 months | 1.14 | 0.94–1.37 | .175 |
| Admissions within 60 days after index | 0.86 | 0.61–1.21 | .386 |
| ED visits within 60 days after index | 1.49 | 1.08–2.07 | .016 |

**Note.** Outcome = any missing PRO among comparison participants only. Because all missing PRO records occurred among comparators, this exploratory model was fit within the comparator subgroup and treatment was not included. Predictors include baseline covariates and post-index utilization and should be interpreted as exploratory associations rather than a causal missingness model.

**Abbreviations:** OR, odds ratio; CI, confidence interval; PRO, patient-reported outcome.

**References**

1. Agency for Healthcare Research and Quality (AHRQ), Healthcare Cost and Utilization Project (HCUP). Fast Stats—National Hospital Utilization and Costs. Accessed October 9, 2025. <https://datatools.ahrq.gov/hcup-fast-stats/>
2. Moore BJ, Liang L, Stocks C. Costs of Treat‑and‑Release Emergency Department Visits in the United States, 2021. HCUP Statistical Brief #311. Agency for Healthcare Research and Quality; 2024. Accessed October 9, 2025. <https://hcup-us.ahrq.gov/reports/statbriefs/sb311-ED-visit-costs-2021.pdf>
3. U.S. Bureau of Labor Statistics. Measuring Price Change in the CPI: Medical care. Updated February 12, 2025. Accessed October 9, 2025. <https://www.bls.gov/cpi/factsheets/medical-care.htm>
4. U.S. Bureau of Labor Statistics. Consumer Price Index for All Urban Consumers: Medical Care (CPIMEDNS). FRED, Federal Reserve Bank of St. Louis. Updated September 11, 2025. Accessed October 9, 2025. <https://fred.stlouisfed.org/series/CPIMEDNS>
5. Agency for Healthcare Research and Quality (AHRQ), Healthcare Cost and Utilization Project (HCUP). HCUP Cost‑to‑Charge Ratio Methodologies. Updated December 17, 2021. Accessed October 9, 2025. <https://hcup-us.ahrq.gov/reports/methods/MS2021-05-CCR-Methodologies.jsp>
6. Agency for Healthcare Research and Quality (AHRQ), Healthcare Cost and Utilization Project (HCUP). HCUP Cost‑to‑Charge Ratio (CCR) for Inpatient Files. Updated April 17, 2025. Accessed October 9, 2025. <https://hcup-us.ahrq.gov/db/ccr/ip-ccr/ip-ccr.jsp>
