# Supplementary material for: Acute‐Care Utilization and Cost Offsets Associated With Language‐Concordant, Pharmacist‐Integrated Care Management Among High‐Need, High‐Cost Adults
Source: Health Serv Res. 2026 May 11;61:e70127. doi: 10.1111/1475-6773.70127 (PMC13160595; doi:10.1111/1475-6773.70127)
Supplement: Supplementary file 2 — Appendix S2: hesr70127‐sup‐0002‐AppendixS2.docx. Table S2. Variable definitions, source fields, coding logic, and validation sources. [file HESR-61-0-s007.docx]

**Table S2**

*Variable Definitions, Source Fields, Coding Logic, and Validation Sources*

| **Domain** | **Variable** | **Source field / code system** | **Codes or value set (version)** | **Algorithm / logic** | **Validation source** |
| --- | --- | --- | --- | --- | --- |
| **Exposure** | Program enrollment | Internal EHR flag | care_mgmt_enrolled ≥ 1 (2024 prod build) | Flag = 1 on or before index discharge date | Internal QA logᵃ |
| **Outcomes** | Hospital admission (all-cause) | Encounter class / UB-04 | EHR encounter_class = "Inpatient" OR claims ClaimType = "IP" (FY 2024) | ≥ 1 qualifying inpatient encounter with admission inside 60-day window | CMS Inpatient Claims Manual |
| **Outcomes** | ED encounter (treat-and-release) | CPT-4 | 99281–99285 (CY 2024) | Outpatient claim with CPT listed AND Discharge_Status ≠ Admit inside 60-day window | NCHS ED utilization specification |
| **Confounders** | Congestive heart failure | ICD-10-CM | I09.9, I11.0, I13.0, I13.2, I25.5, I42.0, I42.5, I42.8, I42.9, I50.x (FY 2024) | ≥ 1 code (any position) in prior 12 mo | Quan 2005 PPV 92% |
| **Confounders** | Chronic pulmonary disease (COPD/asthma) | ICD-10-CM | I27.8–I27.9, J40–J47, J60–J67, J68.4, J70.1, J70.3 (FY 2024) | ≥ 1 code (any position) in prior 12 mo | Quan 2005 PPV 89% |
| **Confounders** | Diabetes mellitus | ICD-10-CM | E10.x–E14.x (FY 2024) | ≥ 1 code (any position) in prior 12 mo | Quan 2005 PPV 96% |
| **Confounders** | Chronic kidney disease | ICD-10-CM | N18.x, N19, Z99.2 (FY 2024) | ≥ 1 code (any position) in prior 12 mo | Quan 2005 PPV 93% |
| **Confounders** | Hypertension | ICD-10-CM | I10.x–I15.x (FY 2024) | ≥ 1 code (any position) in prior 12 mo | Quan 2005 PPV 90% |
| **Confounders** | Depression | ICD-10-CM | F32.x, F33.x (FY 2024) | ≥ 1 code (any position) in prior 12 mo | Quan 2005 PPV 88% |
| **Effect modifiers** | Spanish language preference | EHR language field | "Spanish" (2024 prod build) | Self-reported preferred language = "Spanish" | Analytic codebook |
| **Effect modifiers** | Public insurance (Medicare/Medicaid) | Insurance type field / analytic workbook | Insurance_Type = 2 ("Medicare or Medicaid") | Public insurance indicator used for insurance heterogeneity analyses | Analytic codebook |
| **Effect modifiers** | Social Vulnerability Index (SVI) tertile | CDC/ATSDR SVI 2022 | Continuous SVI mapped to tertiles (2022) | Census tract SVI linked via geocode; tertile cut-points prespecified | CDC/ATSDR SVI documentation |

*Note.* Primary utilization outcomes were defined in symmetric 60-day pre (−60, 0] and post (0, 60] windows unless otherwise stated; baseline comorbidities used a 12-month look-back. Code sets and algorithms were applied identically to enrollees and matched comparators. Table S2 contains manuscript-facing operational definitions and code sets; Appendix S2 provides the machine-readable codebook. “≥” indicates inclusive thresholds; “x” denotes ICD-10-CM code families; continuous SVI was geocoded at the Census-tract level and mapped to prespecified tertiles.

**Abbreviations**: CDC/ATSDR, Centers for Disease Control and Prevention/Agency for Toxic Substances and Disease Registry; CPT-4, Current Procedural Terminology, Fourth Edition; CY, calendar year; EHR, electronic health record; ED, emergency department; FY, fiscal year; ICD-10-CM, International Classification of Diseases, Tenth Revision, Clinical Modification; PPV, positive predictive value; QA, quality assurance; SVI, Social Vulnerability Index; UB-04, Uniform Billing 04.

**References**

Agency for Healthcare Research and Quality, Healthcare Cost and Utilization Project. (2024). *Introduction to the HCUP Nationwide Emergency Department Sample (NEDS), 2022.*

Agency for Toxic Substances and Disease Registry. (2024). *CDC/ATSDR SVI 2022 documentation.*

Centers for Medicare & Medicaid Services. (2024). *Institutional paper claim form (CMS-1450).*

Centers for Medicare & Medicaid Services. (2025). *Medicare claims processing manual: Chapter 3—Inpatient hospital billing* (Rev. 13364).

Quan, H., Sundararajan, V., Halfon, P., Fong, A., Burnand, B., Luthi, J.-C., Saunders, L. D., Beck, C. A., Feasby, T. E., & Ghali, W. A. (2005). Coding algorithms for defining comorbidities in ICD-9-CM and ICD-10 administrative data. *Medical Care, 43*(11), 1130–1139. doi:10.1097/01.mlr.0000182534.19832.83
