# Supplementary material for: Acute‐Care Utilization and Cost Offsets Associated With Language‐Concordant, Pharmacist‐Integrated Care Management Among High‐Need, High‐Cost Adults
Source: Health Serv Res. 2026 May 11;61:e70127. doi: 10.1111/1475-6773.70127 (PMC13160595; doi:10.1111/1475-6773.70127)
Supplement: Supplementary file 3 — Appendix S3: STROBE statement—checklist of items that should be included in reports of observational studies. [file HESR-61-0-s002.docx]

STROBE Statement—checklist of items that should be included in reports of observational studies

|  | Item No. | Recommendation | Page  No. | Relevant text from manuscript |
| --- | --- | --- | --- | --- |
| **Title and abstract** | 1 | (*a*) Indicate the study’s design with a commonly used term in the title or the abstract | 1 | “…Language-Concordant Care Management Reduces Acute-Care Use and Advances Equity: A Retrospective Cohort Study” |
|  |  | (*b*) Provide in the abstract an informative and balanced summary of what was done and what was found | 1 – 2 | Full structured Abstract (Objective, Study Setting and Design, Data Sources and Analytic Sample, Principal Findings, Conclusions) |
| Introduction | | | |  |
| Background/rationale | 2 | Explain the scientific background and rationale for the investigation being reported | 4 | “High-need, high-cost (HNHC) adults… Among these barriers is limited English proficiency (LEP)… acute-care utilization often escalates when insurance status, language preference, and cultural context intersect.” |
| Objectives | 3 | State specific objectives, including any prespecified hypotheses | 6 | “Our objective was to quantify short-horizon utilization and economic effects and examine equity-relevant heterogeneity by language and insurance.” |
| Methods | | | |  |
| Study design | 4 | Present key elements of study design early in the paper | 6 | “We conducted a retrospective cohort study of high-need, high-cost adults discharged from four Phoenix safety-net clinics…” |
| Setting | 5 | Describe the setting, locations, and relevant dates, including periods of recruitment, exposure, follow-up, and data collection | 1; 7 | “…across four safety-net clinics in Phoenix, Arizona (March 2022 – September 2023)…” |
| Participants | 6 | (*a*) *Cohort study*—Give the eligibility criteria, and the sources and methods of selection of participants. Describe methods of follow-up  *Case-control study*—Give the eligibility criteria, and the sources and methods of case ascertainment and control selection. Give the rationale for the choice of cases and controls  *Cross-sectional study*—Give the eligibility criteria, and the sources and methods of selection of participants | 8 | “Between March 2022 and September 2023, we screened 1 452 adults ≥18 y … Of 684 eligible, 512 (74.9 %) were contacted… 16 lacked baseline surveys and were excluded, leaving 263 participants.” |
|  |  | (*b*) *Cohort study*—For matched studies, give matching criteria and number of exposed and unexposed  *Case-control study*—For matched studies, give matching criteria and the number of controls per case | 8 | “From the comparison pool, we selected a 1:1 matched sample via nearest-neighbor matching on prespecified covariates (age, sex, primary language, race/ethnicity, payer, SVI, baseline 12-month admission and ED counts, clinic, index month); observations outside common support were trimmed before propensity-based weighting.” |
| Variables | 7 | Clearly define all outcomes, exposures, predictors, potential confounders, and effect modifiers. Give diagnostic criteria, if applicable | 7-9 | “Primary outcomes were counts of hospital admissions and ED visits … Covariates included age, sex, race, ethnicity, insurance, preferred language, baseline EQ-5D-5L, baseline NPS, prior-year utilization, chronic-disease count, and Social Vulnerability Index tertile…” |
| Data sources/ measurement | 8* | For each variable of interest, give sources of data and details of methods of assessment (measurement). Describe comparability of assessment methods if there is more than one group | 6-7 | “We deterministically linked electronic health-record encounters, claims, HIE alerts, and pharmacist medication-reconciliation logs for all participants… linkage completion rates were 96.6 % for HIE alerts and 95.4 % for reconciliation logs.” |
| Bias | 9 | Describe any efforts to address potential sources of bias | 6 | “E-values were computed to gauge the strength of unmeasured confounding needed to nullify observed effects. Validity checks included entropy balancing, augmented-IPTW DiD, negative-control outcomes, E-values, and Rosenbaum sensitivity bounds.” |
| Study size | 10 | Explain how the study size was arrived at | 13 | “Using power.poisson.test() … produced n = 480 (240 per arm)… the matched analytic sample reached N = 526.” |

Continued on next page

| Quantitative variables | 11 | Explain how quantitative variables were handled in the analyses. If applicable, describe which groupings were chosen and why | 9 | “Secondary outcomes were EQ-5D-5L utilities (range –0.109 to 1.000) and NPS (–100 to +100)… Utilities employed the 2018 US cross-walk, and a minimal clinically important difference (MCID) of ≥ 0.07 was applied; because NPS lacks a validated MCID, changes are interpreted descriptively.” |
| --- | --- | --- | --- | --- |
| Statistical methods | 12 | (*a*) Describe all statistical methods, including those used to control for confounding | 10 | “Analyses followed four sequential steps: (1) propensity-score matching, (2) inverse-probability weighting (IPTW), (3) doubly robust Poisson DiD models producing IRRs and AMEs, and (4) a pre-specified robustness battery.” |
|  |  | (*b*) Describe any methods used to examine subgroups and interactions | 11-12 | “Equity heterogeneity models added insurance, language, and ethnicity interactions, controlling the false-discovery rate with Benjamini–Hochberg; subgroup estimates mirrored overall effects.” |
|  |  | (*c*) Explain how missing data were addressed | 12 | “Directly observed PRO data were available for all 263 enrollees and 224 comparators; the remaining 39 comparator records were retained in the analytic PRO denominator and handled with multiple imputation. Observed-case estimates are IPTW-weighted sensitivity analyses; multiply imputed estimates are the primary analytic PRO results.” |
|  |  | (*d*) *Cohort study*—If applicable, explain how loss to follow-up was addressed  *Case-control study*—If applicable, explain how matching of cases and controls was addressed  *Cross-sectional study*—If applicable, describe analytical methods taking account of sampling strategy | 8 | “Figure S1. Assessment of cohort flow from screening to analysis, four Phoenix safety-net clinics (March 1, 2022–September 30, 2023). A matched comparison group (n=263) was selected, and both groups were analyzed with no loss to follow-up (total N=526).” |
|  |  | (*e*) Describe any sensitivity analyses | 20-22 | “Sensitivity checks—trimming weights, excluding two baseline PRO respondents, and multiple-imputation diagnostics—did not change IRRs or AMEs (≤ 0.001 difference). Five complementary tests supported consistent findings…” |
| Results | | | | |
| Participants | 13* | (a) Report numbers of individuals at each stage of study—eg numbers potentially eligible, examined for eligibility, confirmed eligible, included in the study, completing follow-up, and analysed | 8 | “We screened 1,452 adults… Of 684 eligible, 512 were contacted… 279 consented… 16 excluded… leaving 263; matched to 263 comparators.” |
|  |  | (b) Give reasons for non-participation at each stage | 8 | “Of 1,452 records screened, 684 met high-need, high-cost criteria; 512 were contacted and invited; 106 declined and 127 provided no consent; 279 consented. Sixteen had incomplete baseline patient-reported outcomes, yielding 263 enrolled participants.” |
|  |  | (c) Consider use of a flow diagram | 14 – 15 | Figure 1 Cohort selection flowchart depicting screening, eligibility, enrollment, and analysis populations. |
| Descriptive data | 14* | (a) Give characteristics of study participants (eg demographic, clinical, social) and information on exposures and potential confounders | 17 | Table 1 Baseline equity variables after IPTW (N = 526), showing counts and SMDs for race, ethnicity, language, insurance. |
|  |  | (b) Indicate number of participants with missing data for each variable of interest | 8 | “With no covariate missing > 3 %, we used 20-cycle multiple imputation…” |
|  |  | (c) *Cohort study*—Summarise follow-up time (eg, average and total amount) | 14 | Figure 1 note: “526 Included in Analyses (No loss to follow-up)” |
| Outcome data | 15* | *Cohort study*—Report numbers of outcome events or summary measures over time | 18-19 | “Table 1 summarizes the primary utilization and economic outcomes at 30 and 60 days post-index. For 60-day hospital admissions, IRR = 0.50 (95% CI [0.29, 0.86]; AME = −0.44). For 60-day ED visits, IRR = 0.47 (95% CI [0.28, 0.77]; AME = −0.16). Table S11 reports the formal pre-period lead estimates and joint Wald tests; Table S12 reports the Goodman–Bacon decomposition.” |
|  |  | *Case-control study—*Report numbers in each exposure category, or summary measures of exposure |  |  |
|  |  | *Cross-sectional study—*Report numbers of outcome events or summary measures |  |  |
| Main results | 16 | (*a*) Give unadjusted estimates and, if applicable, confounder-adjusted estimates and their precision (eg, 95% confidence interval). Make clear which confounders were adjusted for and why they were included | 18-19 | Table S4 shows control means, adjusted IRRs (95 % CI), absolute changes, and E-values for ED visits and admissions. |
|  |  | (*b*) Report category boundaries when continuous variables were categorized | n/a | No continuous variables were categorized. |
|  |  | (*c*) If relevant, consider translating estimates of relative risk into absolute risk for a meaningful time period | 18-19 | “…equivalent to 0.44 fewer admissions per participant… 0.16 fewer visits per person.” |

Continued on next page

| Other analyses | 17 | Report other analyses done—eg analyses of subgroups and interactions, and sensitivity analyses | 20-22 | “Sensitivity analyses—entropy balancing, augmented inverse-probability-weighted difference-in-differences, 5th/95th-percentile trimming, multiple-imputation diagnostics, a negative-control outcome, and Rosenbaum bounds—produced convergent estimates... See Table S6 and Figure 2 for subgroup effects, Table S9 for equity-weighted results, and Table S10 for detectable IRRs (80% power).” |
| --- | --- | --- | --- | --- |
| Discussion | | | | |
| Key results | 18 | Summarise key results with reference to study objectives | 22-23 | “Enrollment in a language-concordant, pharmacist-integrated care-management program was associated with 0.44 fewer admissions and 0.16 fewer emergency department visits per participant and $6,421 net standardized savings per enrollee (ROI = 13.66:1) over 60 days.” |
| Limitations | 19 | Discuss limitations of the study, taking into account sources of potential bias or imprecision. Discuss both direction and magnitude of any potential bias | 26 | “Our quasi-experimental design… findings may not generalize… 60-day follow-up window may undervalue later gains… residual confounding remains possible… misclassification of race, ethnicity, or language is possible…” |
| Interpretation | 20 | Give a cautious overall interpretation of results considering objectives, limitations, multiplicity of analyses, results from similar studies, and other relevant evidence | 28 | “In sum, our study demonstrates that tailored, language-concordant care management… substantially lowers acute-care utilization while yielding favorable returns on investment.” |
| Generalisability | 21 | Discuss the generalisability (external validity) of the study results | 26 | “Because the study was conducted in a single urban safety-net system, findings may not generalize to rural or multi-site integrated settings.” |
| Other information | |  | | |
| Funding | 22 | Give the source of funding and the role of the funders for the present study and, if applicable, for the original study on which the present article is based | 30 | “This study received no external or internal funding. Because the work was unfunded, no sponsor influenced the study design….” |

*Give information separately for cases and controls in case-control studies and, if applicable, for exposed and unexposed groups in cohort and cross-sectional studies.

**Note:** An Explanation and Elaboration article discusses each checklist item and gives methodological background and published examples of transparent reporting. The STROBE checklist is best used in conjunction with this article (freely available on the Web sites of PLoS Medicine at http://www.plosmedicine.org/, Annals of Internal Medicine at http://www.annals.org/, and Epidemiology at http://www.epidem.com/). Information on the STROBE Initiative is available at www.strobe-statement.org.
