# Supplementary material for: Acute‐Care Utilization and Cost Offsets Associated With Language‐Concordant, Pharmacist‐Integrated Care Management Among High‐Need, High‐Cost Adults
Source: Health Serv Res. 2026 May 11;61:e70127. doi: 10.1111/1475-6773.70127 (PMC13160595; doi:10.1111/1475-6773.70127)
Supplement: Supplementary file 6 — Figure S1: Assessment of cohort flow from screening to analysis, four Phoenix safety‐net clinics (March 1, 2022–September 30, 2023). Flow of participants from initial screening through inclusion in analyzes. Of 1452 records screened, 684 met high‐need, high‐cost (HNHC) criteria; 512 were contacted and invited; 106 declined and 127 provided no consent; 279 consented. Sixteen had incomplete baseline patient‐reported outcomes (PROs), yielding 263 enrolled participants. A matched comparison group (n = 263) was selected, and both groups were analyzed with no loss to follow‐up (total N = 526). Side boxes enumerate exclusion reasons at each stage (ineligible: < 2 acute encounters; unreachable: ≥ 3 attempts; declined/no consent; incomplete baseline PROs). HNHC was defined a priori as ≥ 2 inpatient admissions or ≥ 4 treat‐and‐release emergency department (ED) visits in the prior 12 months. Matched comparison participants were drawn from the broader eligible usual‐care pool in the same clinics and calendar period, not only from contacted patients who declined or did not provide consent; they were identified from the same source systems, selected 1:1 before IPTW, and screened to confirm no structured care‐management or pharmacist‐counseling exposure. Abbreviations: ADT, admission–discharge–transfer; ED, emergency department; HNHC, high‐need, high‐cost; IPTW, inverse probability of treatment weighting; n, number; PROs, patient‐reported outcomes. Figure S2: Standardized mean differences before and after weighting for baseline covariates (target |SMD| < 0.10). Points show preweighting standardized mean differences (SMDs) and diamonds show postweighting SMDs after stabilized inverse probability of treatment weighting (IPTW) with 1st/99th‐percentile trimming and clinic fixed effects as specified in Methods. The solid vertical line marks 0; dashed lines mark the a priori balance threshold |SMD| < 0.10. Covariates include demographics (age, sex), race/ethnicity (White, Black, Hispani [file HESR-61-0-s005.docx]

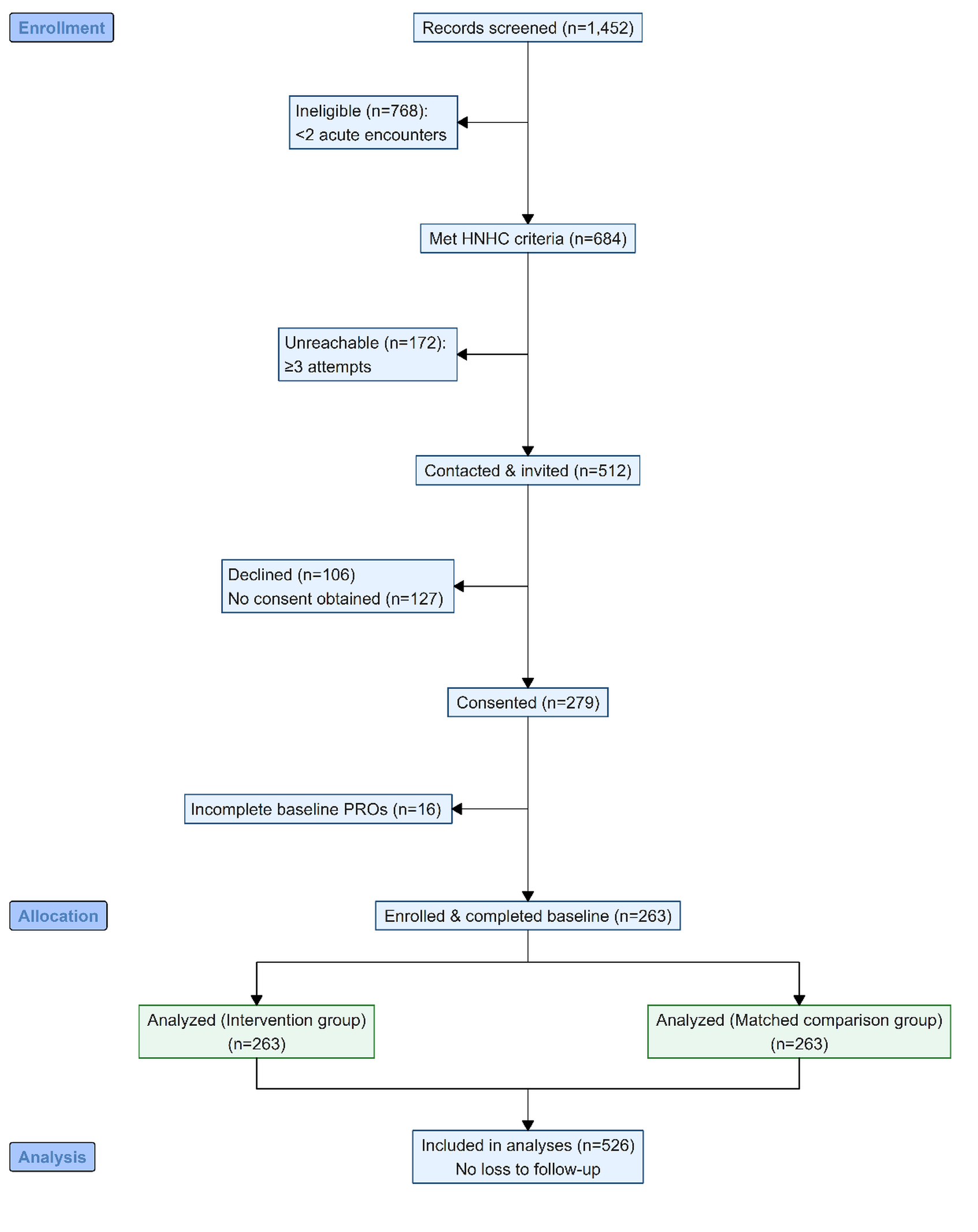


**Figure S1. Assessment of cohort flow from screening to analysis, four Phoenix safety‑net clinics (March 1, 2022–September 30, 2023).**

Flow of participants from initial screening through inclusion in analyses. Of 1,452 records screened, 684 met high‑need, high‑cost (HNHC) criteria; 512 were contacted and invited; 106 declined and 127 provided no consent; 279 consented. Sixteen had incomplete baseline patient‑reported outcomes (PROs), yielding 263 enrolled participants. A matched comparison group (n=263) was selected, and both groups were analyzed with no loss to follow‑up (total N=526). Side boxes enumerate exclusion reasons at each stage (ineligible: <2 acute encounters; unreachable: ≥3 attempts; declined/no consent; incomplete baseline PROs). HNHC was defined a priori as ≥2 inpatient admissions or ≥4 treat‑and‑release emergency department (ED) visits in the prior 12 months. Matched comparison participants were drawn from the broader eligible usual-care pool in the same clinics and calendar period, not only from contacted patients who declined or did not provide consent; they were identified from the same source systems, selected 1:1 before IPTW, and screened to confirm no structured care-management or pharmacist-counseling exposure.
**Abbreviations:** ADT, admission–discharge–transfer; ED, emergency department; HNHC, high‑need, high‑cost; IPTW, inverse probability of treatment weighting; n, number; PROs, patient‑reported outcomes.


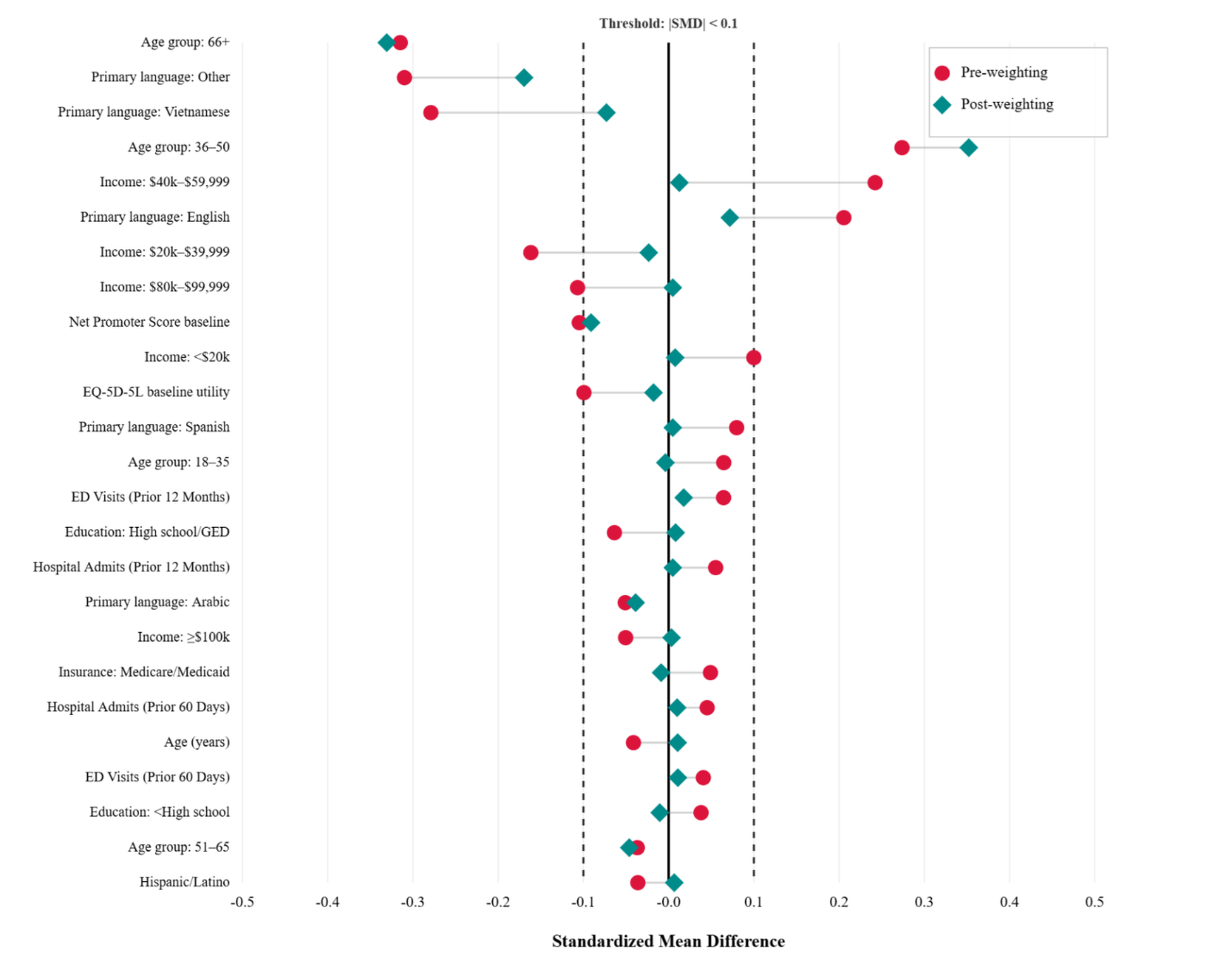


**Figure S2. Standardized mean differences before and after weighting for baseline covariates (target |SMD| < 0.10).**

Points show pre-weighting standardized mean differences (SMDs) and diamonds show post-weighting SMDs after stabilized inverse probability of treatment weighting (IPTW) with 1st/99th-percentile trimming and clinic fixed effects as specified in Methods. The solid vertical line marks 0; dashed lines mark the a priori balance threshold |SMD|<0.10. Covariates include demographics (age, sex), race/ethnicity (White, Black, Hispanic/Latino, Other/multiracial), primary language (English, Spanish, Arabic, Vietnamese, Other), insurance (Medicare/ Medicaid), education, income bands, prior utilization (12-month and 60-day hospital admissions and ED visits), neighborhood Social Vulnerability Index (SVI), and baseline patient-reported measures (EQ-5D-5L utility, Net Promoter Score [NPS]). For continuous variables, SMDs were calculated as the absolute mean difference divided by the pooled standard deviation; for binary indicators, SMDs were calculated using the standard binary form; multicategory variables were represented by level-specific indicators.

**Abbreviations:** ED, emergency department; EQ-5D-5L, EuroQol 5 Dimension 5 Level; IPTW, inverse probability of treatment weighting; NPS, Net Promoter Score; PROs, patient-reported outcomes; SMD, standardized mean difference; SVI, Social Vulnerability Index.


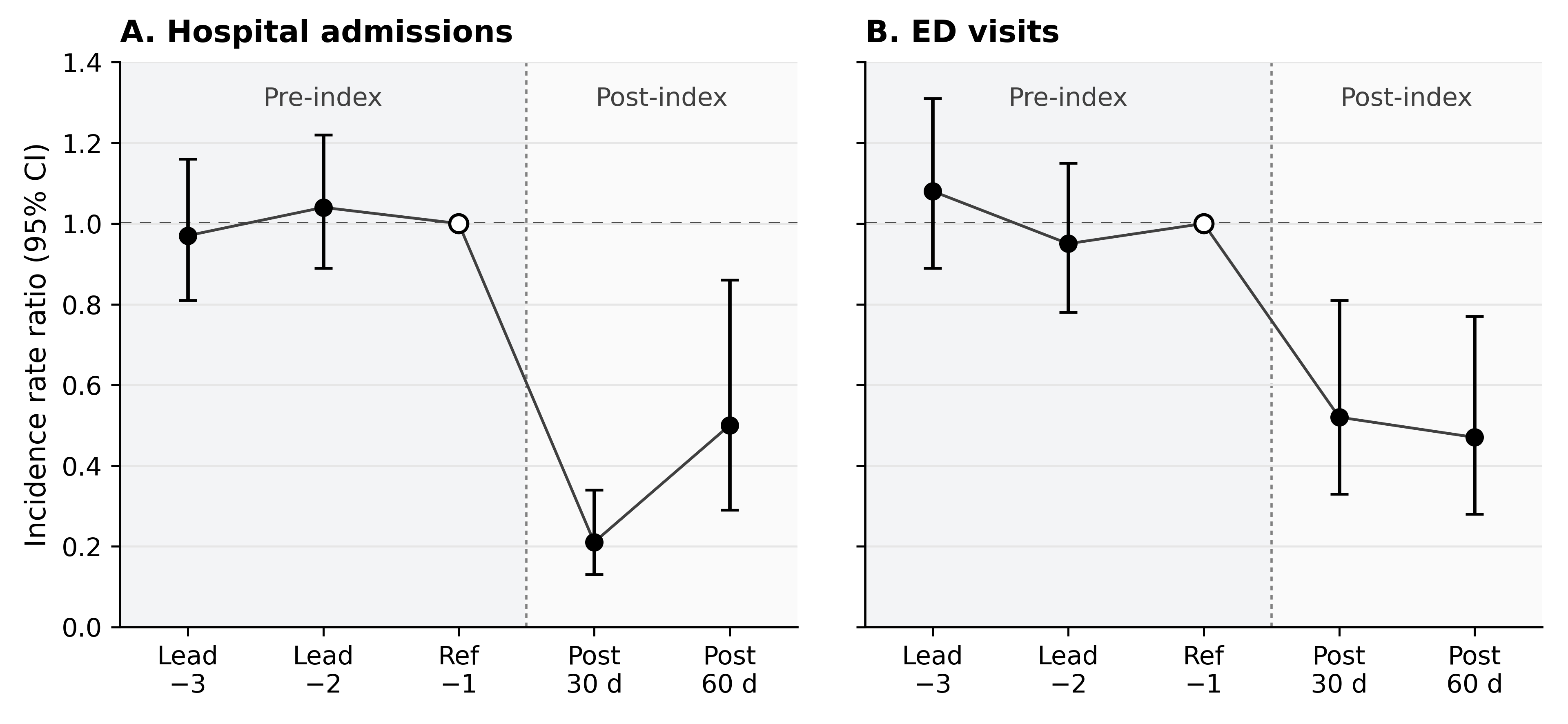


**Figure S3. Event-study-style summary panels for hospital admissions and emergency-department visits.**

These supplemental panels summarize manuscript-reported pre-period lead estimates and post-index incidence-rate ratios for hospital admissions and emergency-department visits, comparing enrollees with weighted usual-care comparators. Figure 1 provides a descriptive summary of mean acute-care use across pre- and post-index windows, and Table S11 reports the formal pre-period lead coefficients and joint Wald tests for both outcomes. Points indicate incidence-rate ratios, error bars indicate 95% confidence intervals, and the dashed reference line marks the null (IRR = 1.00).

**Abbreviations:** CI, confidence interval; ED, emergency department; IRR, incidence-rate ratio.


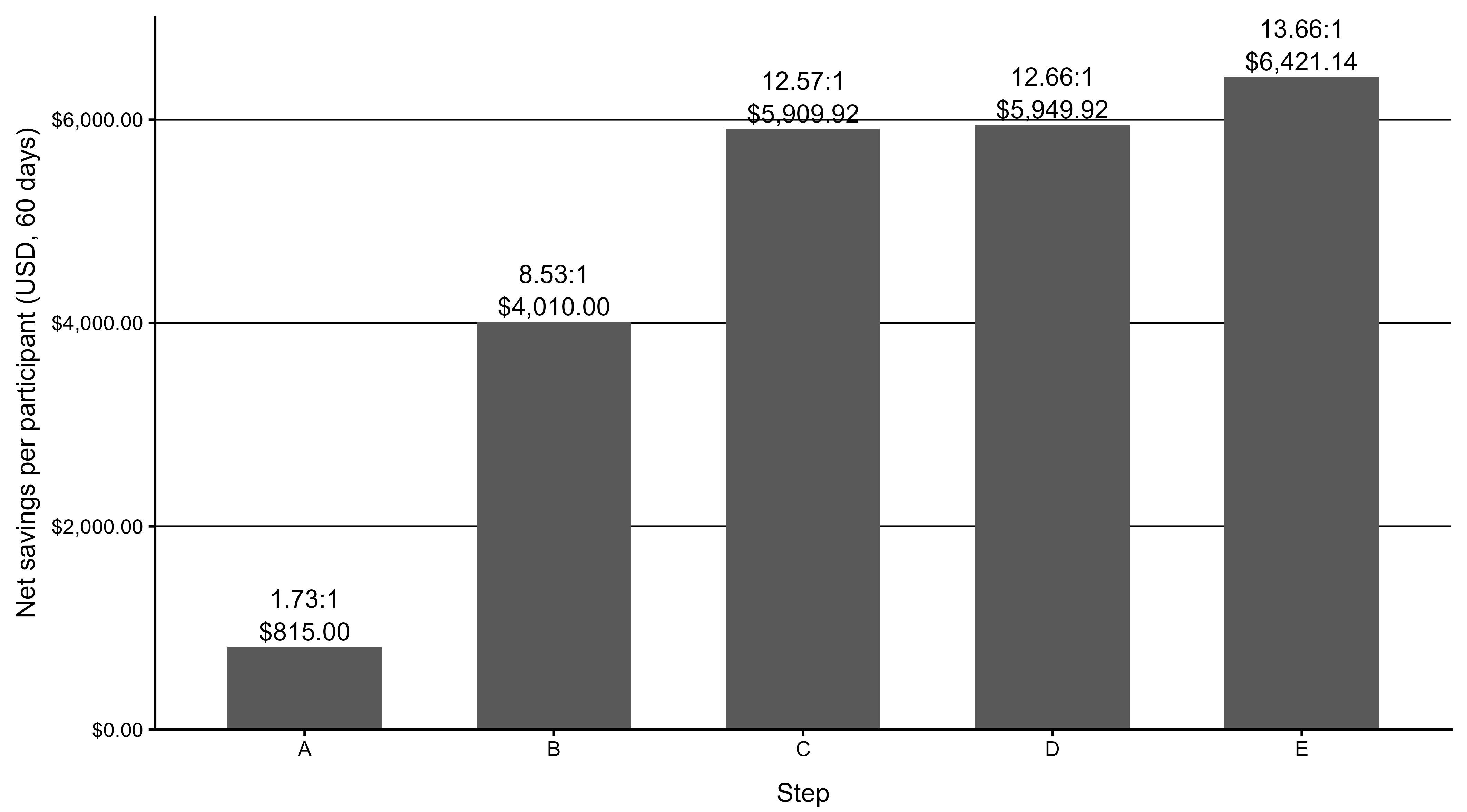


**Figure S4. Step-wise 60-day standardized net savings per enrollee (2024 USD) and ROI.**

Bars show cumulative 60-day standardized net savings per enrollee at each reconciliation step: (A) baseline effects using legacy 2019 unit costs; (B) symmetric duplicate-episode reconciliation with claims backfill applied to both study arms; (C) update of the inpatient unit cost to the HCUP 2021 national mean; (D) update of the treat-and-release emergency-department unit cost to the HCUP 2021 national mean; and (E) repricing of 2021 unit costs to 2024 U.S. dollars using the annual-average Consumer Price Index for All Urban Consumers: Medical Care series (CPIMEDNS). The final bar (E) corresponds to the analytic estimate reported in the manuscript (net savings = $6,421.14; ROI = 13.66:1). ROI was calculated as (standardized costs avoided − program cost) / program cost; program cost was $470 per enrollee. Deduplication and claims backfill are shown only to reconcile source feeds and were not counted as independent value in the ROI calculation. Incremental step contributions and unit-cost inputs are reported in Table S5.

**Abbreviations:** ROI, return on investment; ED, emergency department; HCUP, Healthcare Cost and Utilization Project; CPI-U, Consumer Price Index for All Urban Consumers; CPIMEDNS, CPI-U Medical Care (not seasonally adjusted); USD, U.S. dollars.


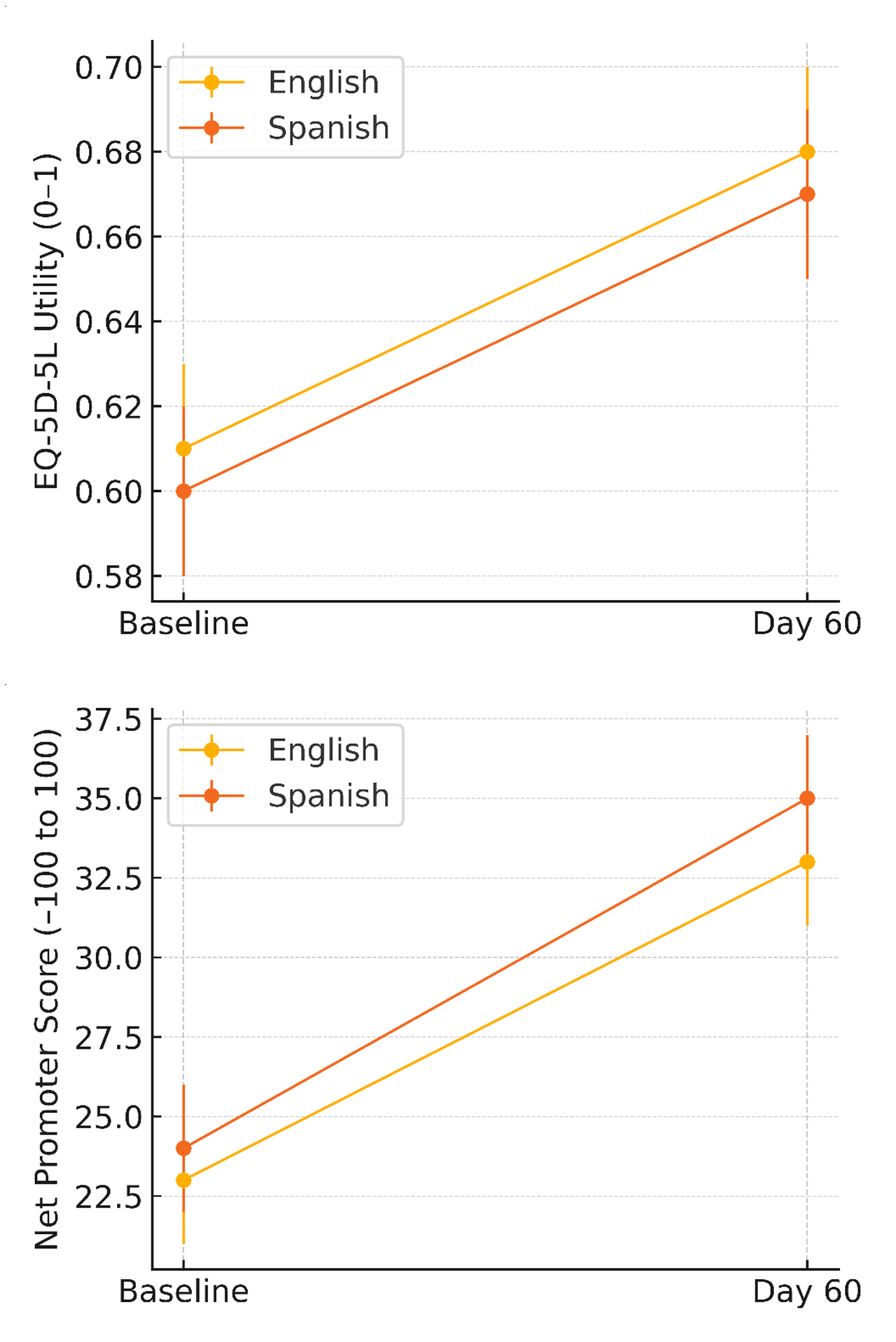


**Figure S5. Longitudinal patient-reported-outcome trends by language preference.**

One panel displays mean EQ-5D-5L utility scores, and the other displays mean Net Promoter Scores, at baseline and approximately 60 days after discharge for Spanish-preferring (red) and English-preferring (orange) enrollees. Error bars indicate 95% confidence intervals. Trends are shown for enrollees only.

**Abbreviations:** CI, confidence interval; EQ‑5D‑5L, EuroQol 5 Dimension 5 Level; NPS, Net Promoter Score; PROs, patient‑reported outcomes.
