# Supplementary material for: Acute‐Care Utilization and Cost Offsets Associated With Language‐Concordant, Pharmacist‐Integrated Care Management Among High‐Need, High‐Cost Adults
Source: Health Serv Res. 2026 May 11;61:e70127. doi: 10.1111/1475-6773.70127 (PMC13160595; doi:10.1111/1475-6773.70127)
Supplement: Supplementary file 7 — Table S1: Duplicate encounter audit and reconciliation (symmetric cross‐feed deduplication + claims backfill) and impact on 60‐day admissions (N = 526). Table S2: Variable definitions, code systems, and algorithms. Table S3: Residual covariate imbalance after IPTW (postweight SMDs; N = 526). Table S4: Predictive enrollment model (baseline‐only) Purpose. Sensitivity analysis to characterize selection into enrollment; does not replace the primary IPTW/DiD causal analysis. Models used only baseline variables (age, sex, Spanish language indicator, public insurance, 12‐month admissions and ED visits, SVI). Reporting follows TRIPOD guidance emphasizing discrimination and calibration (AUC; calibration slope/intercept; decile‐based calibration). Table S5: Step‐wise 60‐day savings and ROI (from dedup/backfill → unit‐cost updates → 2024 repricing). Table S6: Incidence rate ratios for 60‐day outcomes across equity‐related subgroups. Table S7: Baseline characteristics of enrolled care‐management patients and matched comparators before inverse‐probability weighting (N = 526). Table S8: Baseline representativeness of enrollees relative to the broader eligible usual‐care pool. Table S9: Equity‐weighted cost‐effectiveness results (60‐day horizon). Table S10: Detectable incidence‐rate ratios for equity‐related interaction contrasts (60‐day hospital admissions*). Table S11: Formal preperiod lead estimates and joint Wald tests for the parallel‐trends assumption. Table S12: Goodman–Bacon decomposition of the DiD effect (two‐period design). Table S13: Baseline characteristics (Post‐IPTW, ATT; Weighted Means and SDs). Table S14: Decile‐based calibration (GBM model). [file HESR-61-0-s004.docx]

**TABLE S1.** Duplicate encounter audit & reconciliation (symmetric cross‑feed deduplication + claims backfill) and impact on 60‑day admissions (N = 526).

| **Status** | **Enrolled (n events; rate/participant)** | **Comparison (n events; rate/participant)** | **Absolute Δ admissions (per participant)** | **Unadjusted IRR** |
| --- | --- | --- | --- | --- |
| Before deduplication/backfill | 53; 0.2015 | 84; 0.3194 | −0.1179 | 0.6310 |
| After symmetric deduplication and claims backfill | 53; 0.2015 | 96; 0.3650 | −0.1635 | 0.5521 |

*Note:* Absolute Δ = (enrolled rate − comparison rate); negative values favor the program. Unadjusted IRR assumes equal 60‑day person‑time per arm (263 participants × 60 days), computed as the ratio of event counts: before, IRR = 53/84 = 0.6310; after, IRR = 53/96 = 0.5521. The “after” row reflects symmetric ±24‑hour episode deduplication across feeds with precedence order claims→EHR→HIE and concurrent claims backfill, which added +12 counted inpatient episodes to the comparison arm and 0 to the enrolled arm over 60 days; denominators (N = 526; 263 per arm) match the manuscript cohort. ROI accounting in the economic analysis does not credit deduplication; only corrected effect sizes are used in cost calculations (see main text and supplement).

**Abbreviations:** IRR, incidence rate ratio; ROI, return on investment; EHR, Electronic Health Record; HIE, Health Information Exchange.

**TABLE S2.** Variable Definitions, Code Systems, and Algorithms.

| **Domain** | **Variable** | **Code system** | **Codes/Value set** | **Version** | **Algorithm/Logic** |
| --- | --- | --- | --- | --- | --- |
| **Exposure** | Program enrolment | Internal EHR flag | Binary field care_mgmt_enrolled ≥ 1 | 2024 prod build | Flag = 1 on or before index discharge date |
| **Outcomes** | Hospital admission (all cause) | Encounter class / UB‑04 | EHR encounter_class = “Inpatient” OR claims ClaimType = “IP” | FY 2024 | ≥ 1 qualifying inpatient encounter with admission inside window |
|  | ED encounter (treat and release) | CPT‑4 | 99281–99285 | CY 2024 | Outpatient claim with CPT listed AND Discharge_Status ≠ Admit inside 60‑day window |
| **Confounders** | Congestive heart failure | ICD‑10‑CM | I09.9, I11.0, I13.0, I13.2, I25.5, I42.0, I42.5, I42.8, I42.9, I50.x | FY 2024 | ≥ 1 code (any position) in prior 12 mo |
|  | Chronic pulmonary disease (COPD/asthma) | ICD‑10‑CM | I27.8–I27.9, J40–J47, J60–J67, J68.4, J70.1, J70.3 | FY 2024 | ≥ 1 code (any position) in prior 12 mo |
|  | Diabetes mellitus | ICD‑10‑CM | E10.x–E14.x | FY 2024 | ≥ 1 code (any position) in prior 12 mo |
|  | Chronic kidney disease | ICD‑10‑CM | N18.x, N19, Z99.2 | FY 2024 | ≥ 1 code (any position) in prior 12 mo |
|  | Hypertension | ICD‑10‑CM | I10.x–I15.x | FY 2024 | ≥ 1 code (any position) in prior 12 mo |
|  | Depression | ICD‑10‑CM | F32.x, F33.x | FY 2024 | ≥ 1 code (any position) in prior 12 mo |
| **Effect modifiers** | Spanish language preference | EHR language field | “Spanish” | 2024 prod build | Self‑reported preferred language = “Spanish” |
|  | Medicaid coverage | Insurance type field | “Medicaid” | 2024 prod build | Primary or secondary payer = Medicaid on index discharge |
|  | Social Vulnerability Index (SVI) tertile | CDC SVI 2022 | Continuous SVI mapped to tertiles | 2022 | Census tract SVI linked via geocode; tertile cut‑points pre‑specified |

*Note:* Outcomes were measured in the 60‑day post‑discharge window; baseline comorbidities used a 12‑month look‑back. Code sets: ICD‑10‑CM (FY 2024), CPT‑4 (CY 2024), and UB‑04 billing classifications; SVI corresponds to the CDC/ATSDR 2022 tract‑level index. Algorithms were prespecified and applied identically to enrolled and comparator cohorts. “≥” indicates inclusive thresholds; “x” denotes ICD‑10‑CM code families.

**Abbreviations:** CPT, Current Procedural Terminology; EHR, electronic health record; ED, emergency department; ICD‑10‑CM, International Classification of Diseases, 10th Revision, Clinical Modification; SVI, Social Vulnerability Index; UB‑04, Uniform Billing.

**TABLE S3.** Residual Covariate Imbalance After IPTW (post‑weight SMDs; N = 526).

| **Covariate** | **Type** | **SMD (unweighted)** | **SMD (weighted)** | **\|SMD\| (weighted)** | **Threshold** | **Exceeds 0.10** |
| --- | --- | --- | --- | --- | --- | --- |
| Age (years) | Contin. | 0.0073 | -0.0156 | 0.0156 | 0.1 | No |
| Admissions in prior 12 months | Contin. | -0.0503 | -0.0430 | 0.0430 | 0.1 | No |
| ED visits in prior 12 months | Contin. | 0.0644 | -0.0120 | 0.0120 | 0.1 | No |
| Social Vulnerability Index (SVI) | Contin. | -0.0123 | 0.0095 | 0.0095 | 0.1 | No |
| Sex: Female | Binary | 0.0076 | -0.0026 | 0.0026 | 0.1 | No |
| Race: White | Binary | 0.0190 | -0.0380 | 0.0380 | 0.1 | No |
| Race: Black or African American | Binary | -0.0114 | -0.0359 | 0.0359 | 0.1 | No |
| Race: American Indian or Alaska Native | Binary | -0.0076 | 0.0575 | 0.0575 | 0.1 | No |
| Race: Asian | Binary | -0.0038 | 0.0077 | 0.0077 | 0.1 | No |
| Race: Native Hawaiian or Other Pacific Islander | Binary | 0.0038 | 0.0087 | 0.0087 | 0.1 | No |
| Ethnicity: Hispanic or Latino | Binary | 0.0141 | -0.0184 | 0.0184 | 0.1 | No |
| Ethnicity: Not Hispanic or Latino | Binary | 0.0076 | 0.0348 | 0.0348 | 0.1 | No |
| Ethnicity (categorical, (3‑level; omnibus) | Categorical (3‑level; omnibus) | -0.0010 | 0.0096 | 0.0096 | 0.1 | No |
| Insurance type: Private | Binary | -0.0028 | 0.0134 | 0.0134 | 0.1 | No |
| Insurance type: Medicare or Medicaid | Binary | 0.0038 | 0.0075 | 0.0075 | 0.1 | No |
| Insurance type (categorical, 3‑level; omnibus) | Categorical (3‑level; omnibus) | 0.0063 | -0.0053 | 0.0053 | 0.1 | No |
| Language: English | Binary | 0.0913 | -0.0001 | 0.0001 | 0.1 | No |
| Language: Spanish | Binary | 0.0266 | 0.0186 | 0.0186 | 0.1 | No |
| Language: Arabic | Binary | -0.0000 | -0.0035 | 0.0035 | 0.1 | No |
| Language: Chinese | Binary | -0.0076 | 0.0282 | 0.0282 | 0.1 | No |
| Language: Vietnamese | Binary | -0.0570 | -0.0243 | 0.0243 | 0.1 | No |
| Language: Other | Binary | -0.0532 | -0.0189 | 0.0189 | 0.1 | No |

*Note:* Standardized mean differences (SMDs) were calculated post-weighting using pooled SD for continuous covariates and the standard binary form. We used stabilized IPTW (ATT; GBM) with 1st/99th-percentile trimming and renormalization; the prespecified imbalance threshold was SMD ≥0.10.

**Abbreviations:** SMD, standardized mean difference; IPTW, inverse probability of treatment weighting; GBM, gradient‑boosted machines; ATT, average treatment effect in the treated; BH FDR, Benjamini–Hochberg false‑discovery rate.

**TABLE S4. Predictive enrollment model (baseline‑only)**

**Purpose.** Sensitivity analysis to characterize selection into enrollment; does not replace the primary IPTW/DiD causal analysis. Models used only baseline variables (age, sex, Spanish language indicator, public insurance, 12‑month admissions and ED visits, SVI). Reporting follows TRIPOD guidance emphasizing discrimination and calibration (AUC; calibration slope/intercept; decile‑based calibration).

| **Model** | **AUC (bootstrap 95% CI)** | **Brier score†** | **Calibration intercept (95% CI)** | **Calibration slope (95% CI)** | **Overall event rate (enrollment)** |
| --- | --- | --- | --- | --- | --- |
| Logistic regression | 0.70 (0.66–0.74) | 0.188 | +0.01 (−0.04 to +0.06) | 0.98 (0.90–1.06) | 0.408 (279/684) |
| Gradient‑boosted trees (GBM) | 0.74 (0.70–0.78) | 0.182 | −0.02 (−0.07 to +0.03) | 1.02 (0.93–1.09) | 0.408 (279/684) |

†Lower Brier indicates better overall accuracy (calibration‑sensitive). Definitions and rationale in clinical prediction literature.

**TABLE S5.** Step‑wise 60‑day savings and ROI (from dedup/backfill → unit‑cost updates → 2024 repricing).

| **Step** | **Change applied** | **Δ Admissions (per participant)** | **Δ ED visits (per participant)** | **Unit cost (USD)**** | **Gross 60‑day savings (USD)** | **Net savings* (USD)** | **ROI (×:1)** |
| --- | --- | --- | --- | --- | --- | --- | --- |
| **A** | Baseline (original effect sizes; 2019 unit costs) | 0.12 | 0.17 | 10,000 / 500 | 1,285.00 | 815.00 | 1.73 |
| **B** | Apply symmetric deduplication + claims backfill (Model‑adjusted AMEs) | 0.44 | 0.16 | 10,000 / 500 | 4,480.00 | 4,010.00 | 8.53 |
| **C** | Update inpatient unit cost (HCUP 2021 mean) | — | — | 14,318 / 500 | 6,379.92 | 5,909.92 | 12.57 |
| **D** | Update ED unit cost (HCUP 2021 mean) | — | — | 14,318 / 750 | 6,419.92 | 5,949.92 | 12.66^a^ |
| **E** | Reprice 2021 unit costs to 2024 USD using CPIMEDNS annual‑average ratio (≈ 1.073) | — | — | 15,369.19 / 805.06 | 6,891.14 | 6,421.14 | 13.66^b^ |

*** Net savings = Gross − program cost ($470 per enrollee). Order is admission / ED.

**Abbreviations:** CPIMEDNS, Consumer Price Index for All Urban Consumers: Medical Care (not seasonally adjusted); ED, emergency department; FRED, Federal Reserve Economic Data; HCUP, Healthcare Cost and Utilization Project; ROI, return on investment; USD, U.S. dollars.

^a^ Exact unrounded ROI for Step D: 12.6594:1.

^b^ 2024 USD repricing uses the CPI medical-care CPIMEDNS annual average 2024/2021 ratio; exact values depend on the finalized 2024 annual average (method and series documented by FRED/BLS). Inpatient national mean cost for 2021 from HCUP Fast Stats (AHRQ); treat-and-release ED unit cost from Roemer M. *Costs of Treat-and-Release Emergency Department Visits in the United States, 2021.* HCUP Statistical Brief No. 311. Rockville, MD: Agency for Healthcare Research and Quality; September 2024. Available at: <https://hcup-us.ahrq.gov/reports/statbriefs/sb311-ED-visit-costs-2021.pdf>. Accessed October 9, 2025. Repricing to 2024 USD uses BLS CPI—Medical Care (CPIMEDNS) annual averages (not seasonally adjusted), retrieved from FRED, Federal Reserve Bank of St. Louis: <https://fred.stlouisfed.org/series/CPIMEDNS> (accessed October 9, 2025).

**TABLE S6.** Incidence rate ratios for 60‑day outcomes across equity‑related subgroups.

| **Subgroup** | **60‑day ED visits IRR (95% CI)** | **60‑day hospital admissions IRR (95% CI)** |
| --- | --- | --- |
| **Race/Ethnicity** |  |  |
| Black | 0.58 (0.41–0.81) | 0.29 (0.17–0.50) |
| Hispanic/Latino | 0.60 (0.38–0.94) | 0.33 (0.16–0.70) |
| White | 0.65 (0.46–0.91) | 0.36 (0.22–0.58) |
| Other/multiracial | 0.70 (0.48–1.03) | 0.35 (0.18–0.69) |
| **Sex** |  |  |
| Female | 0.60 (0.43–0.85) | 0.31 (0.19–0.52) |
| Male | 0.64 (0.42–0.97) | 0.34 (0.18–0.63) |
| **Baseline 60‑day ED use** |  |  |
| ≥ 1 prior ED visit | 0.55 (0.42–0.72) | 0.28 (0.18–0.43) |
| 0 prior ED visits | 0.71 (0.49–1.03) | 0.36 (0.23–0.58) |
| **Language preference** |  |  |
| Spanish‑preferring | 0.42 (0.18–0.97) | 0.36 (0.16–0.81) |
| English‑preferring | 0.45 (0.32–0.64) | 0.38 (0.26–0.56) |
| Other languages | 1.36 (0.45–4.10) | 0.21 (0.10–0.42) |
| **Insurance type** |  |  |
| Public insurance (Medicaid/Medicare) | 0.69 (0.45–1.05) | 0.40 (0.27–0.59) |
| Private insurance | 0.34 (0.21–0.54) | 0.23 (0.14–0.38) |

*Note:* IRRs < 1.00 indicate lower post‑index utilization in the enrolled group relative to comparison within the stated subgroup. 95% CIs/p‑values reflect BH FDR for interaction analyses and Holm adjustment for prespecified subgroup endpoints; statistical significance is assessed by 95% CIs not crossing 1.00 (ED outcomes not significant for Other/multiracial, 0 prior ED visits, Other languages, and Public insurance). Subgroup estimates are from stratified analyses within the IPTW cohort (treated = 263; weighted controls = 263).

**Abbreviations:** CI, confidence interval; ED, emergency department; IRR, incidence rate ratio; IPTW, inverse probability of treatment weighting

**TABLE S7. Baseline characteristics of enrolled care-management patients and matched comparators before inverse-probability weighting (N = 526)**

| **Characteristic** | **Enrolled (n = 263)** | **Comparison (n = 263)** | **Std. diff** |
| --- | --- | --- | --- |
| Age, y (mean ± SD) | 52.1 ± 19.8 | 52.0 ± 22.0 | 0.01 |
| **Age groups** |  |  |  |
| 18–35 y | 91 (34.6%) | 83 (31.6%) | — |
| 36–50 y | 58 (22.1%) | 31 (11.8%) | — |
| 51–65 y | 60 (22.8%) | 58 (22.1%) | — |
| ≥66 y | 54 (20.5%) | 91 (34.6%) | — |
| Female sex | 155 (58.9%) | 153 (58.2%) | 0.02 |
| **Race/Ethnicity¹** |  |  |  |
| White | 57 (21.7%) | 49 (18.6%) | 0.08 |
| Black | 66 (25.1%) | 70 (26.6%) | 0.03 |
| Hispanic/Latino | 53 (20.2%) | 55 (20.9%) | 0.02 |
| Other/multiracial¹ | 87 (33.1%) | 89 (33.8%) | 0.02 |
| Medicare/Medicaid insurance | 140 (53.2%) | 139 (52.9%) | 0.01 |
| **60-day history** |  |  |  |
| ED visits (mean ± SD) | 0.8 ± 1.5 | 0.8 ± 1.4 | 0.01 |
| Hospital admits (mean ± SD) | 0.3 ± 0.5 | 0.3 ± 0.5 | 0.02 |
| **12-month history** |  |  |  |
| ED visits (mean ± SD) | 1.5 ± 2.0 | 1.4 ± 2.0 | 0.06 |
| Hospital admits (mean ± SD) | 1.2 ± 2.0 | 1.3 ± 2.0 | 0.05 |
| **Patient-reported outcomes²** |  |  |  |
| EQ-5D-5L (mean ± SD) | 0.61 ± 0.10 | 0.62 ± 0.11 | 0.10 |
| Net Promoter Score (mean ± SD) | 19.5 ± 4.5 | 20.0 ± 5.3 | 0.10 |
| Social Vulnerability Index (mean ± SD) | 0.68 ± 0.18 | 0.69 ± 0.18 | 0.01 |

Notes. Continuous variables are mean ± SD; categorical are n (%). Std. diff (absolute standardized difference): continuous = |μ₁−μ₀| / √[(σ₁²+σ₀²)/2]; categorical = |p₁−p₀| / √[p̄(1−p̄)], where p̄ = (p₁+p₀)/2. Values > 0.10 denote meaningful imbalance.

Abbreviations: ED, emergency department; EQ-5D-5L, EuroQol 5-Dimension 5-Level; NPS, Net Promoter Score; SD, standard deviation; SVI, Social Vulnerability Index.

¹ “Other/multiracial” is a mutually exclusive aggregate category used for reporting and small-cell suppression (≤10): American Indian/Alaska Native, Asian, Native Hawaiian/Other Pacific Islander, multiracial, and “unknown/declined.” Race/ethnicity categories in this table are mutually exclusive: White (non-Hispanic), Black (non-Hispanic), Hispanic/Latino (any race), Other/multiracial (non-Hispanic).

² Baseline PRO rows are based on directly observed baseline PROs (enrolled n=263; comparison n=224). Primary PRO analyses retained all 263 comparison participants through multiple imputation; see Appendix S1, Table A1.

**TABLE S8** Baseline representativeness of enrollees relative to the broader eligible usual-care pool

| **Variable (baseline)** | **Enrolled (n=279)** | **Eligible-not-enrolled (n=405)** | **\|SMD\|** |
| --- | --- | --- | --- |
| Age, years (mean±SD) | 52.2±19.5 | 54.4±19.0 | 0.11 |
| Female, n (%) | 165 (59.1%) | 219 (54.1%) | 0.10 |
| Primary language: Spanish, n (%) | 86 (30.8%) | 105 (25.9%) | 0.11 |
| Insurance: Medicare/Medicaid (public), n (%) | 148 (53.0%) | 198 (48.9%) | 0.08 |
| 12-month hospital admissions (mean±SD) | 1.20±2.00 | 1.05±1.90 | 0.08 |
| 12-month ED visits (mean±SD) | 1.50±2.00 | 1.40±2.10 | 0.05 |
| Social Vulnerability Index (SVI) (mean±SD) | 0.68±0.18 | 0.64±0.19 | 0.22 |

*Note.* *Enrolled* = participants who consented to the program (n = 279). *Eligible-not-enrolled* = patients who met eligibility during the same clinics and study period but did not enroll (n = 405). This table characterizes the broader eligible usual-care source population from which matched comparators were later drawn; it documents source-population comparability rather than study-sample balance; matched pre-weight characteristics are shown in Table S7, and post-IPTW balance is shown in Table S3 and Figure S2. Baseline measures were taken from the EHR, claims, and health information exchange; patient-reported outcomes were not used. Standardized mean differences (SMDs) compare the 2 groups: for continuous variables, SMD is the difference in group means divided by the pooled standard deviation; for binary variables, SMD is the difference in group proportions divided by the standard deviation of the pooled proportion. Values with |SMD| ≥ 0.10 are interpreted as meaningfully different and are shown in bold. Abbreviations: ED, emergency department; SVI, Social Vulnerability Index from CDC/ATSDR tract-level linkage.

**Representativeness of enrollees.** Among 684 eligible patients, enrollees (n = 279) were slightly younger (52.2 vs 54.4 years; SMD = 0.11), more often Spanish-preferring (31% vs 26%; SMD = 0.11), and resided in more vulnerable neighborhoods (SVI 0.68 vs 0.64; SMD = 0.22); differences in payer mix and prior utilization were <0.10. These modest differences suggest that enrollees arose from a source population broadly comparable to the eligible usual-care pool, with residual selection most evident for age, Spanish preference, and neighborhood vulnerability.

**TABLE S9.** Equity‑weighted cost‑effectiveness results (60‑day horizon).

| **Race / Ethnicity subgroupᵃ** | **Participants, n** | **Δ Cost (USD)** | **Δ Admissions avoided** | **ICER (USD / admission)ᵇ** | **Equity weight ω** | **Net monetary benefit (NMB)ᶜ** | **Equity‑weighted NMB (ω × NMB)** |
| --- | --- | --- | --- | --- | --- | --- | --- |
| Black | 66 | –2,150 | 0.22 | Dominant | 1.20 | $4,350 | $5,220 |
| Hispanic / Latino | 53 | –1,800 | 0.18 | Dominant | 1.15 | $3,600 | $4,140 |
| White | 57 | –1,650 | 0.15 | Dominant | 1.00 | $3,150 | $3,150 |
| Other/multiracialᵈ | 87 | –1,200 | 0.12 | Dominant | 1.10 | $2,400 | $2,640 |
| Overall | 263 | –6,421 | 0.44 | Dominant | — | $10,821 | — |

*Note:* Overall row uses the AME‑based (model‑based) estimand consistent with the main manuscript economics and the Step‑E reconciliation (2024 USD): Δ Cost (net) = (0.44 × inpatient unit cost [2024] + 0.16 × ED unit cost [2024]) − $470 program cost = –$6,421, with Δ Admissions = 0.44; NMB = –Δ Cost + λ × Δ Admissions = $6,421 + (10,000 × 0.44) = $10,821 (λ=$10,000/admission avoided). Subgroup rows reproduce the published subgroup estimates in this table; equity weighting multiplies the NMB only (ω×NMB), not costs or outcomes.

ᵃCells with n < 11 are aggregated to preserve confidentiality.

ᵇICER = Δ Cost / Δ Admissions; “Dominant” = lower cost and fewer admissions vs comparator, so ICER not required.

ᶜNMB = –Δ Cost + λ × Δ Admissions.

ᵈOther/Multiracial includes Asian, Native Hawaiian/Other Pacific Islander, and multiracial.

**Abbreviations.** ED, emergency department; ICER, incremental cost‑effectiveness ratio; NMB, net monetary benefit.

**Unit‑cost sources and repricing.** Inpatient national mean cost (2021) from HCUP Fast Stats/NIS and treat‑and‑release ED unit cost (2021) from HCUP Statistical Brief #311; both repriced to 2024 USD using CPI‑U Medical Care (CPIMEDNS; annual‑average 2024/2021 ratio); program cost $470 per enrollee (see Supplementary Table S5/Figure S4; Economic Methods).

**TABLE S10.** Detectable incidence‑rate ratios for equity‑related interaction contrasts (60‑day hospital admissions*).

| **Interaction term** | **Total Nᵃ** | **Baseline admission rateᵇ** | **Detectable IRR (80% power)ᶜ** | **Relative reduction** |
| --- | --- | --- | --- | --- |
| Language × Medicaid | 181 | 0.293 | 0.33 | 67% |
| Language × Public insurance (Medicaid + Medicare) | 238 | 0.400 | 0.49 | 51% |
| Language × Private insurance | 213 | 0.489 | 0.51 | 49% |
| Black race | 136 | 0.400 | 0.34 | 66% |
| Hispanic/Latino ethnicity | 108 | 0.382 | 0.25 | 75% |
| Non‑Hispanic ethnicity | 418 | 0.716 | 0.69 | 31% |

*Note:* ᵃNumber of analytic participants (enrolled + comparison) in each stratum after propensity‑score matching. ᵇMean 60‑day inpatient‑admission count per comparison‑arm participant within the stratum. ᶜSmallest IRR (< 1.00) detectable with 80% power (two‑sided α = .05) for a two‑sample Poisson comparison assuming equal group sizes; normal‑approximation method in R (power.poisson.test). *Hospital admissions occurring within 60 days of index discharge.

**Abbreviations:** ED, emergency department. IRR = incidence rate ratio.

**TABLE S11.** Formal pre-period lead estimates and joint Wald tests for the parallel-trends assumption

| **Outcome** | **Pre-period contrast (reference *t* = −1)** | **IRR** | **95% CI** | ***p*** | **Joint Wald test** | **Joint *p*** |
| --- | --- | --- | --- | --- | --- | --- |
| 60-day hospital admissions | Lead *t* = −3 vs. *t* = −1 | 0.97 | [0.81, 1.16] | .73 | — | — |
| 60-day hospital admissions | Lead *t* = −2 vs. *t* = −1 | 1.04 | [0.89, 1.22] | .61 | — | — |
| 60-day hospital admissions | Joint Wald test of all pre-period leads | — | — | — | χ²(2) = 0.58 | .75 |
| 60-day ED visits | Lead *t* = −3 vs. *t* = −1 | 1.08 | [0.89, 1.31] | .44 | — | — |
| 60-day ED visits | Lead *t* = −2 vs. *t* = −1 | 0.95 | [0.78, 1.15] | .59 | — | — |
| 60-day ED visits | Joint Wald test of all pre-period leads | — | — | — | χ²(2) = 1.21 | .55 |

*Note:* Reference period is *t* = −1. Joint Wald tests evaluate the null of no differential pre-trend across the reported pre-period leads. Em dashes indicate not applicable.

**Abbreviations:** CI = confidence interval; ED = emergency department; IRR = incidence-rate ratio.

**TABLE S12.** Goodman–Bacon Decomposition of the DiD Effect (two‑period design).

| **Outcome** | **Weight (ω)** | **Component effect (ln IRR)** | **IRR (overall DiD)** | **Share of total weight (%)** |
| --- | --- | --- | --- | --- |
| Admissions (60d) | 1.0 | -0.6989 | 0.4971 | 100 |
| ED visits (60d) | 1.0 | -0.7615 | 0.4670 | 100 |

*Note:* With a single treatment cohort and two periods, the decomposition reduces to one comparison (ω = 1); the component equals ln(IRR) from the Poisson DiD.

**Abbreviations:** DiD, difference in differences; IRR, incidence rate ratio.

**TABLE S13.** Baseline Characteristics (Post‑IPTW, ATT; Weighted Means and SDs).

| **Variable** | **Comparison (N = 263) Mean** | **Comparison SD** | **Enrolled (N = 263) Mean** | **Enrolled SD** |
| --- | --- | --- | --- | --- |
| Age (years) | 52.5 | 18.7 | 52.1 | 19.8 |
| Sex (coded) | 1.6 | 0.5 | 1.6 | 0.5 |
| Primary language (coded) | 1.4 | 1.0 | 1.4 | 0.9 |
| Race (coded) | 2.0 | 1.2 | 2.1 | 1.2 |
| Ethnicity (coded) | 1.8 | 0.4 | 1.8 | 0.4 |
| Insurance type (coded) | 1.5 | 0.5 | 1.5 | 0.5 |
| Social Vulnerability Index (SVI) | 0.7 | 0.2 | 0.7 | 0.2 |
| Admissions in prior 12 months | 1.3 | 1.8 | 1.2 | 2.0 |
| ED visits in prior 12 months | 1.6 | 2.2 | 1.5 | 2.0 |

*Note:* Weighted means and standard deviations are shown on the original coded scale after stabilized IPTW (ATT); categorical variables remain numeric codes, so balance should be interpreted primarily from Table S3 and Figure S2.

**Abbreviations:** ATT, average treatment effect on the treated; IPTW, inverse probability of treatment weighting; SD, standard deviation.

**TABLE S14. Decile‑based calibration (GBM model)**

| **Risk decile** | **N** | **Predicted *p*(enroll)** | **Observed enrollment** | **Observed rate** |
| --- | --- | --- | --- | --- |
| 1 (lowest) | 68 | 0.16 | 10 | 0.147 |
| 2 | 68 | 0.22 | 14 | 0.206 |
| 3 | 68 | 0.28 | 20 | 0.294 |
| 4 | 68 | 0.32 | 23 | 0.338 |
| 5 | 68 | 0.36 | 26 | 0.382 |
| 6 | 68 | 0.40 | 28 | 0.412 |
| 7 | 68 | 0.45 | 31 | 0.456 |
| 8 | 68 | 0.49 | 33 | 0.485 |
| 9 | 69 | 0.55 | 37 | 0.536 |
| 10 (highest) | 69 | 0.78 | 57 | 0.826 |

*Interpretation.* Discrimination is moderate (AUC ≈ 0.74), and calibration is near‑ideal (slope ≈ 1, intercept ≈ 0) with monotonic observed rates across deciles; the highest‑risk decile captures 57/279 (20%) of enrollees. Calibration constructed on n = 682 complete‑case adults (two with missing predictors excluded); observed counts remain 279.
